# Loss of Brd4 alleviates pathological bone loss via Slc9b2 suppression in osteoclastogenesis

**DOI:** 10.1002/ctm2.70496

**Published:** 2025-10-20

**Authors:** Xiaohe Wang, Fangji Luo, Guiqiang Miao, Boyuan Zheng, Chenhao Xu, Vincent Kam Wai Wong, Yuanshu Peng, Rong Zeng, Jinzhu Pang, Xuguang Zhang, Zhenyu Ju, Zhengang Zha, Xiaogang Wang, Xiaofei Zheng, Huan‐Tian Zhang

**Affiliations:** ^1^ Department of Bone and Joint Surgery The First Affiliated Hospital of Jinan University & Key Laboratory of Regenerative Medicine of Ministry of Education Jinan University Guangzhou Guangdong China; ^2^ Department of Orthopedics The First Affiliated Hospital of University of Science and Technology of China Hefei Anhui China; ^3^ Department of Orthopedics, Foshan Fosun Chancheng Hospital Foshan Clinical Medical School of Guangzhou University of Chinese Medicine Foshan Guangdong China; ^4^ Dr. Neher's Biophysics Laboratory for Innovative Drug Discovery State Key Laboratory of Mechanism and Quality of Chinese Medicine Macau University of Science and Technology Macao SAR China; ^5^ Department of Materials Science and Engineering College of Chemistry and Materials Jinan University Guangzhou Guangdong China; ^6^ Mengniu Institute of Nutrition Science Global R&D Innovation Center, Inner Mongolia Mengniu Dairy (Group) Shanghai Institute of Nutrition and Health the Chinese Academy of Sciences Shanghai China; ^7^ Department of Orthopedics, Guangdong Provincial Key Laboratory of Bone and Joint Degenerative Diseases the Third Affiliated Hospital of Southern Medical University Guangzhou Guangdong China

**Keywords:** Brd4, osteoclastogenesis, osteoporosis, Slc9b2

## Abstract

**Background:**

Epigenetic regulation plays a crucial role in skeletal degenerative diseases, including osteoporosis. As an epigenetic reader, bromodomain protein 4 (Brd4) is known as a key driver of gene activation; however, its role in maintaining skeletal homeostasis remains largely unknown.

**Methods:**

We examined Brd4 expression in bone specimens from osteoporotic patients and mouse models, and generated two types of Brd4 conditional knockout mice using Lyz2‐Cre and Ctsk‐Cre systems. Bone mass, osteoclast differentiation, and metabolic activity were assessed under physiological and pathological conditions, including ovariectomy and lipopolysaccharide (LPS) challenge. Mechanistic analyses were performed using transcriptomic screening, gene overexpression, and pharmacological interventions.

**Results:**

Brd4 expression was markedly elevated in bones from osteoporotic patients and mice compared with normal controls. Deletion of Brd4 increased basal bone mass and prevented bone loss induced by ovariectomy or LPS, primarily by suppressing osteoclastogenesis through inhibition of glycolysis. Unbiased screening identified solute carrier family 9 member B2 (Slc9b2) as a downstream effector of Brd4. Overexpression of Slc9b2 partially rescued the impaired osteoclastogenesis caused by Brd4 depletion. Moreover, phosphatidylserine‐containing nanoliposomes loaded with Brd4‐targeting PROTACs (e.g., dBET6) effectively suppressed osteoclastogenesis and alleviated pathological bone loss.

**Conclusions:**

Brd4 serves as a crucial regulator of osteoclast metabolism and differentiation. Targeting Brd4 represents a promising therapeutic strategy for the prevention and treatment of osteoporosis and pathological bone loss.

**Key points:**

- Brd4 is highly expressed in osteoporotic patients and animals.
- Brd4 is crucial for glycolysis‐mediated OC differentiation.
- The loss of Brd4 in bone marrow monocytes or osteoclasts increases basal bone mass and prevents pathological bone loss.
- Slc9b2 is a novel target of Brd4 in mediating osteoclastogenesis.
- Targeting Brd4 by dBET6@PSLs could alleviate osteoporosis progression.

## Graphical abstract

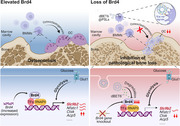

## 1 INTRODUCTION

Bone mass and metabolism are regulated by continuous bone remodelling, maintaining a balance between bone formation by osteoblast (OB) and bone resorption by osteoclast (OC).^1^ Degenerative diseases like osteoporosis are characterised by the deterioration of bone microarchitecture, resulting in low bone density and increased fragility due to excessive bone resorption caused by either an increased number or exaggerated activities of OC.^2^, ^3^ OC are differentiated from the monocyte/macrophage lineage of haematopoietic cells and function by organising their actin cytoskeleton and the secretion of acidic hydrolase cathepsin K.^3^ Osteoclastogenesis is an energy‐intensive process that requires a significant metabolic shift towards increased glycolysis.^4^ However, the upstream signals that fine‐tune these processes, particularly from the perspective of epigenetic regulation during osteoclastogenesis, remain largely unknown.

An increasing number of studies have revealed that OC differentiation and activity are controlled by epigenetic mechanisms, largely through regulating the accessibility of transcriptional machinery to key OC genes.^5^, ^6^ Among these mechanisms, the bromodomain and extra‐terminal domain (BET) family of proteins, including BRD2, BRD3, BRD4 and BRDT, has been reported to play crucial roles in gene transcription.^7^, ^8^ BET proteins are key regulators in the maintenance of skeletal health, and their dysregulation, particularly Brd4, has been implicated in a series of degenerative skeletal diseases such as osteoporosis.^8^, ^9^, ^10^ For instance, Brd4 facilitates the osteogenic differentiation of human bone marrow mesenchymal stem cells (MSCs) through pathways like the Wnt/β‐catenin signalling,^11^ and treatment with BET inhibitors (BETi) results in reduced expression of a series of OB/osteocyte‐specific genes.^12^ On the other hand, inhibition of Brd4 by several BETi prevents OC differentiation by interfering with Brd4‐dependent RANKL activation of *Nfatc1* transcription, thereby suppressing bone destruction in various inflammatory diseases, including arthritis, bone tumour and osteoporosis.^13^ Brd4 is also demonstrated to suppress osteoclastogenesis through positively regulating other key transcription factors, including c‐Fos and NF‐κB.^14^, ^15^ However, its precise role in osteoclastogenesis and pathological bone loss is not yet fully determined.

Anti‐resorptive therapies, such as bisphosphonates and denosumab, have become the first‐line treatments for osteoporosis.^2^, ^16^ Recently, targeted drug delivery systems, particularly those leveraging nanotechnology, have been extensively utilised in clinical settings due to their favourable attributes such as biocompatibility, biodegradability and versatility^17^, ^18^ and have offered promising solutions by enhancing bone targeting efficacy.^19^ We have recently utilised proteolysis‐targeting chimeras (PROTACs) to degrade Brd4, thereby reprogramming macrophage polarisation for degenerative diseases.^9^ This study found that Brd4 expression was elevated in bone samples from osteoporotic patients and mice. Brd4 loss suppressed OB differentiation and impaired osteoclastogenesis. Conditional knockout of *Brd4* increased basal bone mass and prevented bone loss caused by ovariectomy (OVX) and lipopolysaccharide (LPS). Unbiased screening revealed Slc9b2 as a downstream target of Brd4, necessary for osteoclastogenesis. Additionally, targeting Brd4 with phosphatidylserine (PS)‐containing nanoliposomes (PSLs) loaded with dBET6 suppressed osteoclastogenesis and alleviated bone loss. These findings suggest Brd4 as a promising target for preventing osteoporosis and pathological bone loss.

## 2 MATERIALS AND METHODS

### 2.1 Human bone tissue collection

All experimental procedures received approval from the Ethics Committee of the First Affiliated Hospital of Jinan University (Ethical approval number: KY‐2021‐065). Bone mineral density (BMD) was assessed using dual‐energy X‐ray absorptiometry and classified into three categories: normal (femoral neck (FN) BMD T‐score > −1.0), osteopenia (−2.5 < FN‐BMD T‐score ≤ −1.0) and osteoporosis (FN‐BMD T‐score ≤ −2.5). Participants with other metabolic disorders or abnormalities in any of the screening laboratory tests (including complete blood count, serum calcium, phosphorus, albumin, etc.) were excluded from the study. Detailed participant characteristics are provided in Table S1. All participants were informed about the study's purpose and provided written informed consent prior to surgery. Bone specimens were obtained from patients with osteoarthritis undergoing total knee arthroplasty at the First Affiliated Hospital of Jinan University.

### 2.2 Animals

Heterozygous recombinant mice carrying the *Brd4 (Brd4^f/+^)* genotype (Strain NO. TOS181224DA2) and having a C57BL/6 genetic background were sourced from Cyagen Biosciences (Guangzhou, China). *Lyz2‐Cre* mice (Strain NO. T003822) and *Ctsk‐Cre* mice (Strain NO. T006950), both with a C57BL/6 genetic background, were obtained from GemPharmatech (Jiangsu, China). The mice were housed in a specific pathogen‐free environment with a constant ambient temperature of 22 ± 2°C, humidity of 55 ± 10% and a 12‐h light/dark cycle. All procedures involving animals were approved by the Institutional Animal Care and Use Committee of Jinan University (Ethical approval number: IACUC‐20230102‐05) and adhered to the guidelines outlined in the ‘Guide for the Care and Use of Laboratory Animals’ published by the National Institute of Health in China. *Brd4^f/f^* mice were generated by crossing the targeted heterozygous mice and used as wild‐type (WT) control. Subsequently, *Lyz2‐Cre; Brd4^f/f^* and *Ctsk‐Cre; Brd4^f/f^* mice were generated by crossing *Brd4^f/f^* mice with *Lyz2‐Cre* or *Ctsk‐Cre* mice, respectively, followed by genotyping. The PCR genotyping primers used are listed in Table S3.

### 2.3 Data acquisition and correlation analysis

Pre‐aligned mouse and human single‐cell RNA sequencing (scRNA‐seq) matrix files were acquired from GEO datasets GSE145477 (mouse bone marrow), GSE147287 (human femoral head), GSE169396 (human femoral head) and DISCO (Deeply Integrated human Single‐Cell Omics data.^20^ The standard Seurat pipeline^21^ was used for filtering, normalisation, variable gene selection, dimensionality reduction analysis and clustering. For the integrated dataset, batch integration was performed using Harmony (version 1.0).^22^ Cell types were annotated according to the metadata from published datasets.^23^, ^24^ DEGs between young and aged groups were identified using FindMarkers within Seurat, with *p* values calculated by default setting. MAGIC^25^ was used to impute the drop‐out values for visualisation.

### 2.4 RNA extraction and RT‐PCR quantification

Total RNA purification, cDNA reverse transcription and quantitative PCR were performed according to the protocol we described previously.^26^ Relative gene expression was normalised to GAPDH, and primers for other genes were listed in Table S4.

### 2.5 Immunoblotting

Immunoblotting was carried out as described before.^27^ Briefly, an equal amount of proteins (20 µg) was electrophoresed by SDS‐PAGE gel (6–12%), and proteins were then transferred to the polyvinylidene fluoride (0.22 mM, PALL‐BSP0161) membranes. After blocking with 5% skim milk, the membranes were incubated overnight at 4°C with primary antibody against Brd4 (BETHYL; A700‐004, 1:1000), Nfatc1 (Cell Signaling Technology; 14074, 1:1000), GAPDH (Cell Signaling Technology; 2118, 1:1000), Ctsk (Abcam; ab19027, 1:1000), c‐Fos (Abcam; ab222699, 1:500), Acp5 (Abcam; ab235448, 1:500) and Slc9b2 (Immunoway; YN8225, 1:1000). This was followed by incubation with HRP‐conjugated secondary antibodies (1:1000) at room temperature for 1 h. The immunoreactive bands were visualised using Clarity Western ECL Substrate (Bio‐Rad, Hercules, CA).

### 2.6 Cell culture and OC induction

Raw264.7 cells (ATCC, USA) were cultured and maintained as previously described.^28^ For OC induction, Raw264.7 cells were treated with 100 ng/mL RANKL for 5 d, with media changes conducted every 2 days. For chondrocytes culture, cartilage from mouse knee joint was isolated and digested with 10 mL 0.25% trypsin‐ethylenediaminetetraacetic acid (EDTA) (Thermo Fisher Scientific) at 37°C for 30 min, followed by the digestion with 2 mg/mL type I collagenase (Thermo Fisher Scientific) at 37°C for another 4 h. After centrifugation, the pellet was resuspended with Dulbecco's modified Eagle medium supplemented with 10% heat‐inactivated FBS (Life Technologies, CA, USA), 100 units/mL penicillin (Life Technologies) and 100 µg/mL streptomycin (Life Technologies). Bone marrow cells were first isolated from 4 to 6‐week‐old mice possessing *Brd4^f/f^*, *Lyz2‐Cre; Brd4^f/f^* or *Ctsk‐Cre; Brd4^f/f^* genotypes, respectively. Then, cells were cultured in α‐MEM supplemented with 30 ng/mL M‐CSF (MCE, HY‐P70553) for 4 d to generate bone marrow macrophages (BMMs). For OC induction, BMMs were stimulated with 30 ng/mL M‐CSF and 100 ng/mL RANKL (R&D, 462‐TEC‐010) for a minimum of 5 d, and the control group was treated with 30 ng/mL M‐CSF and an equal volume of PBS corresponding to that of RANKL.

### 2.7 Histological staining

For the histological evaluation, knee joint and femur bone tissues were initially fixed in 4% paraformaldehyde (PFA) for 48 h and subsequently decalcified in diethylpyrocarbonate‐treated EDTA solution. After decalcification, the specimens were then embedded in paraffin and sectioned to a thickness of 5 µm. Haematoxylin and eosin (H&E) staining of the sections was performed as previously described.^28^ For IHC staining, paraffin sections underwent a process of dewaxing and hydration, followed by antigen retrieval. The sections were then incubated with primary antibodies against Brd4 (1:100; BETHYL; A700‐004, USA), Runx2 (1:100; Abcam; ab236639, UK) and Col10a1 (Invitrogen; 14‐9771‐80, 1:50) at 4°C overnight. Subsequently, the sections were incubated with secondary antibodies and subjected to DAB colour development (Cell Signaling Technology; 8509P, USA). Images were captured under a light microscope (390335; Leica, Germany). For IF staining, frozen sections were incubated overnight at 4°C with primary antibodies against Brd4 (BETHYL; A700‐004, 1:200), Runx2 (Abcam; ab236639, 1:100), Sox9 (#82630; Cell Signaling Technology; 1:100), F4/80 (Servicebio; GB113373, 1:400), Slc9b2 (Immunoway; YN8225, 1:100) and Ctsk (Abcam; ab19027, 1:100), respectively. This was followed by incubation with Alexa Fluor 488‐conjugated (#4408S; Cell Signaling Technology; 1:200) or Alexa Fluor 555‐conjugated (#4413S; Cell Signaling Technology; 1:200) secondary antibodies. DAPI (#0483; Cell Signaling Technology; 0.5 µg/mL) was used for nuclear counterstaining. Additionally, some of the paraffin sections were examined by IF using the TSAPlus Kit (Servicebio; G1236; China) according to the manufacturer's protocols. Images were acquired under a laser scanning confocal microscope (Zeiss LSM 880, Germany).

### 2.8 OVX‐induced osteoporosis mouse model

To establish the OVX model, 12‐week‐old female mice with the *Brd4^f/f^* genotype, as well as *Lyz2‐Cre; Brd4^f/f^* and *Ctsk‐Cre; Brd4^f/f^* genotypes, were randomly divided into two groups: the sham‐operated group and the OVX group. In the sham‐operated group, the ovaries were simply exteriorised and repositioned. In the OVX group, osteoporotic bone loss was induced by performing bilateral OVX under sodium pentobarbital‐induced anaesthesia. The surgical incisions in the muscle and skin were carefully closed using 6‐0 silk sutures. After an 8‐week period of OVX modelling, all mice were euthanised, and uteri were observed to confirm the osteoporotic status. Femurs were collected for micro‐CT analysis to assess bone density and structure, followed by the histological examination.

### 2.9 LPS‐induced osteoporosis mouse model

For the LPS model, 12‐week‐old male mice with the *Brd4^f/f^* genotype and the *Lyz2‐Cre; Brd4^f/f^* genotypes were randomly assigned into two groups: the PBS group and the LPS group, with six mice in each group. The LPS group received intraperitoneal injections of LPS (Beyotime Biotechnology; ST1470) at a dose of 5 mg/kg on day 0 and day 4. On day 8, bone specimens were collected from the mice for micro‐CT analysis based on the protocol we established previously.^28^

### 2.10 TRAP staining

Cells and mouse knee sections were fixed and stained to assess the presence of OC using a commercial kit following the manufacturer's instructions (Sigma–Aldrich; 387A). OC were identified as cells with more than five nuclei. TRAP‐positive cells were visualised, and the number of OC as well as the size of OC were measured using ImageJ software (National Institutes of Health, Bethesda, MD, USA).

### 2.11 F‐actin ring formation assay

Cells were fixed with 4% PFA for 15 min at room temperature, followed by staining in the dark for 2 h with Actin‐Tracker Red‐555 (Beyotime; C2203S; China), according to the manufacturer's instructions. After three washes with PBS, cells were incubated with DAPI for 10 min in darkness, followed by three additional washes with PBS. Representative images were acquired using a laser scanning confocal microscope (Zeiss LSM 880, Germany). Image processing and analysis were performed using ImageJ software.

### 2.12 Bone resorption assays

BMMs were plated (250,000 cells/well) in 96‐well plates, previously equipped with bone slices (IDS, DT‐1BON1000‐96‐1). Cells were cultured in appropriate media for at least 14 days at 37°C. After this time, cells were washed twice with PBS and removed from bone slices via ultrasonication in 250 µL of 70% isopropanol for at least 15 min at high power. Resorption pit formation was visualised by 100 µL toluidine blue (1%; dissolved in water) staining for 2 min at room temperature. The slices were then rinsed with 250 µL PBS at least five times to wash out residues. Resorption pits are now stained in dark blue and resorption area can be quantified via ImageJ software or resorption pits/well can be counted via light microscopy.

### 2.13 Seahorse XF96 glycolysis stress assay

The extracellular acidification rate (ECAR) was assessed using the XF96 extracellular flux analyser (Seahorse Bioscience, Billerica, MA, USA). Briefly, Raw264.7 cells were plated at a density of 3 × 10^4^/well in Seahorse XF96 plates and treated with varying concentrations of dBET6 for 24 h, with or without OC induction for 5 d. Additionally, BMMs from *Lyz2‐Cre; Brd4^f/f^* and *Brd4^f/f^* mice were seeded at a density of 1 × 10^5^/well and subjected to OC differentiation for 5 d. The assay was conducted following previously established protocols.^29^ All Seahorse flux data were normalised to protein content obtained using an automated cell counter.

### 2.14 Micro‐CT scanning and analysis

Following euthanasia, the right femurs were harvested from the mice and fixed in 4% PFA for 48 h. Then, they were preserved in 70% ethanol. High‐resolution ex vivo micro‐CT imaging was conducted using SkyScan1176 system (Bruker micro‐CT, Kontich, Belgium) to acquire images, which were subsequently transformed into 2D representations. The region spanning 0.1 mm to 1.0 mm above the femur growth plate was reconstructed to facilitate 3D visualisation. Morphometric parameters were quantitatively analysed using suitable software. The morphometric indices evaluated included BV/TV, Tb.Th, Tb.N, Tb.Sp, cortical BV/TV and BMD.

### 2.15 RNA‐Seq analysis

Total RNA was isolated from BMMs derived from *Brd4^f/f^* or *Lyz2‐Cre; Brd4^f/f^* mice, treated with or without OC induction for a period of 5 d. The quality of the extracted RNA was assessed using an Agilent Bioanalyzer 4150 system (Agilent Technologies, CA, USA). Subsequently, cDNA libraries were constructed using VAHTS Universal V6 RNA‐seq Library Prep Kit for Illumina (Vazyme), in accordance with the manufacturer's instructions. Genes that exhibited a |log_2_ (fold change)| > 1 and adjusted *p* value < 0.05 were considered to be significantly DEGs, as determined using the DESeq2 R package. Heat maps of altered genes were generated using HISAT2 software. To further understand the biological functions of these genes, the identified DEGs were analysed by gene ontology and KEGG pathways enrichment using clusterProfiler R software package.

### 2.16 Lentiviral transduction

The lentivirus for Cre gene overexpression was purchased from OBiO Technology (Shanghai) Corp., Ltd. This lentiviral vector system, incorporating EGFP and the Cre gene, was engineered using a synthetic Cre gene inserted into the pLenti‐EF1‐EGFP‐P2A‐puro‐CMV plasmid. Transductions were done in 24‐well plates. Lentivirus vectors for Cre gene overexpression (multiplicity of infection [MOI] = 100) were added to OB isolation from 3‐day‐old *Brd4^f/f^* mice.^30^ 24 h later, the lentivirus was removed, and an osteogenic medium was added. Similarly, the Slc9b2 gene overexpression lentivirus was procured from GenePharma Inc (Shanghai, China), constructed with the pGLV5/GFP/Puro lentiviral vector plasmid to generate stable clones. Transductions were done in six‐well plates. Cells were seeded at 2.5 × 10^5^ per well for 24 h. Then, they were infected by applying viral supernatant (MOI = 80) in 2.0 mL of FBS‐free medium for another 24 h. An osteoclastogenic medium was replaced for 5 d.

### 2.17 Preparation and characterisation of dBET6@PSLs

dBET6@PSLs were synthesised using thin‐film hydration, ultrasonication and extrusion.^9^, ^31^ A mixture of 49 mg Soybean phosphatidylcholine (SPC), 11 mg 1, 2‐dipalmitoyl‐sn‐glycero‐3‐phospho‐L‐serine (PS), 8 mg cholesterol and 0.04 mg dBET6 was dissolved in 6 mL chloroform/ethanol (5:1). After solvent evaporation at 37°C, the lipid film was hydrated with PBS (pH 7.4) by shaking and vortexing for 30 min at 52°C, yielding a 10 mM lipid concentration. The solution was probe‐sonicated (150 W) for 5 min on ice and extruded through a 100 nm membrane four times. Control PSLs without dBET6 were prepared using the same method. The particle size, polydispersity index (PDI) and zeta potential of both PSLs and dBET6@PSLs were characterised using a Zetasizer (Nano‐ZS; Malvern Instruments, Malvern, UK). Encapsulation efficiency (EE) and drug loading efficiency (DLE) of dBET6 were calculated as we described before.^9^ The dBET6 content was quantified via UV‐Vis spectrophotometry (Persee TU‐1810SPC, China) at a wavelength of 218 nm. The morphology of the liposomes was examined using transmission electron microscopy (TEM). The degree of PS exposure on the liposome surface was evaluated by binding Annexin V‐FITC (Key GEN BioTECH, KGA105, China) to PS, as described in our previous research.^9^

### 2.18 In vitro drug release and the stability of dBET6@PSLs

The release of dBET6 from the liposomes was assessed using the dialysis bag method in PBS‐ethanol (7:3, v/v). Briefly, 2 mL of dBET6@PSLs solution was placed in a dialysis bag with an 8000–12 000 Da cutoff. The bags were immersed in 300 mL of PBS‐ethanol (7:3, v/v) at 37°C with a paddle rotation speed of 100 rpm. At predetermined intervals, 2 mL of the medium was withdrawn for analysis, and an equivalent volume of fresh medium was added. The dBET6 concentration in the samples was measured by UV‐Vis spectrophotometry (Persee TU‐1810SPC) at 218 nm. Additionally, long‐term physical stability of the liposomes was tested by storing freshly prepared liposomal suspensions at 4°C for 3 weeks. The zeta potential, particle size and PDI of the liposomes were monitored throughout the storage period as previously described.^9^

### 2.19 Retention and biodistribution of dBET6@PSLs

Mice with a C57BL/6 background were randomised divided into three groups for intramedullary injection into the femur with distinct formulations: DiR, PSLs/DiR and dBET6@PSLs/DiR. Each formulation was labelled with DiR at a concentration of 0.06 mM. Fluorescence distribution within the femoral marrow cavity was visualised at 1, 3, 5 and 7 d post‐injection using an imaging system (IVIS Lumina XRMS Series III; PerkinElmer). For biodistribution analysis, formulations were intramedullary injected into mice and major organs (heart, liver, spleen, lung and kidney) as well as joint tissues were harvested for ex vivo imaging 1 day post‐euthanasia. Radiant efficiency of major organs and joint tissues was quantified following previous studies.^9^

### 2.20 Treatment with dBET6@PSLs

12‐week‐old male C57BL/6 mice purchased from GemPharmatech (Nanjing, China) were randomly allocated to four experimental groups: intraperitoneal injection of PBS (healthy group), intraperitoneal injection of LPS (osteoporosis group), femoral intramedullary injection of dBET6@PSLs (125 µM of the total lipid concentration, containing 50 nM dBET6) or dBET6@PSLs (500 µM of the total lipid concentration, containing 200 nM dBET6), followed by LPS injection. Briefly, anaesthesia was induced by intraperitoneal injection of pentobarbital sodium. Surgery was performed on the right hindlimb of all animals via a skin incision to expose the patellar tendon. The surrounding tissue were dissected to expose the distal femoral shaft. A 26G needle was used to cannulate the intramedullary space, and the compounds were delivered to the proximal femur with an insulin needle. Bone wax was applied and the incision was closed. The first injection of LPS or PBS occurred immediately after the operation, followed by the same intervals. All experiments were approved by the local ethical committee of Jinan University (Ethical approval number: IACUC‐20240329‐11).

### 2.21 Statistical analyses

Data are expressed as means ± standard deviation. For comparisons between two groups, a two‐tailed Student's *t*‐test was employed. One‐way analysis of variance (ANOVA) was used for comparisons among multiple groups. All statistical analyses were performed using GraphPad Prism 10 software. Significance levels were set at *p < .05; **p < .01; ***p < .001; ****p < .0001; n.s., not significant.

## 3 RESULTS

### 3.1 Brd4 expression and its correlation with osteoporotic status during aging

**FIGURE 1:**
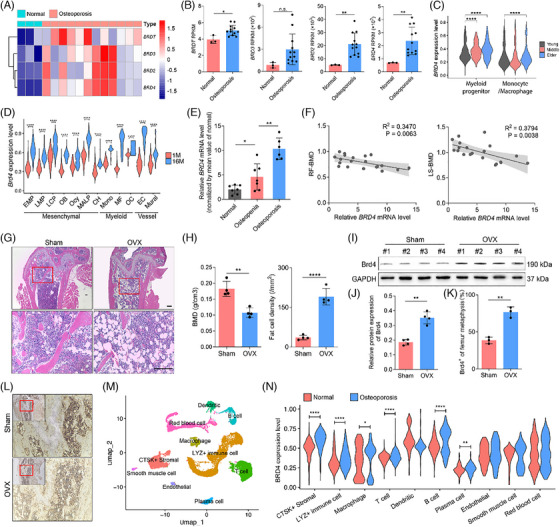
BRD4 expression is elevated in the osteoporotic patients and animals. (A) Heatmap illustrating expression profiles of *BRD* gene family (*BRDT*, *BRD3*, *BRD2* and *BRD4*) in the femoral head of normal and osteoporosis patients. (B) The expression levels of *BRDT*, *BRD2*, *BRD3* and *BRD4* obtained from GSE230665 were quantified. (C) Violin plot presented the *BRD4* expression in human bone marrow monocyte lineage cells (DISCO). (D) Violin plot presented the *Brd4* expression in mouse bone marrow mesenchymal lineage cells, endothelial cells, mural cells and monocyte lineage cells (GSE145477). (E) qRT‐PCR analysis reveals differential *BRD4* mRNA expression in distal femur specimens from patients with varying bone mineral densities (BMD), classified as normal (*n* = 7), osteopenia (*n* = 7) and osteoporosis (*n* = 6). (F) Correlation analysis between the *BRD4* mRNA expression and the BMD measurements at the right femur (RF‐BMD) and the lumbar spine (LS‐BMD). (G) Representative images of H&E staining of distal femur bone of sham group and OVX group. (H) BMD and fat cells density of distal femur bone of sham group and OVX group (*n* = 4). (I and J) Immunoblotting analysis of the Brd4 protein expression in the femur of 5‐month‐old sham‐operated or OVX mice (*n* = 4). (K and L) Representative images of immunohistochemistry staining of Brd4 in the femur metaphysis, with the quantification of Brd4^+^ cells (*n* = 3). (M) Uniform Manifold Approximation and Projection identified 10‐cell clusters in the bone marrow cells of osteoarthritis and osteoporosis patients. Each cluster is represented by a different colour. (N) Violin plot showing the elevated *BRD4* expression in bone marrow cells of osteoporosis patients, compared with osteoarthritis patients. Comparisons in (F) were conducted using simple linear regression. Comparisons in the others were conducted by Student's *t*‐test, two‐tailed. *p < .05, **p < .01, ****p < .0001, n.s., not significant.

Recent studies have emphasised the significance of BET proteins, including BRDT, BRD3, BRD2 and BRD4, in bone biology.^7^, ^10^ Analysis of data from GSE230665 revealed a significant increase in mRNA levels of these genes in femoral cancellous bone from osteoporotic patients compared with those with normal BMD (Figure 1A,B). Our previous research, in conjunction with other studies, has demonstrated the pivotal role of *Brd4* in both skeletal development and aging‐related diseases.^8^, ^9^ Next, the expression of Brd4 in aged clinical specimens was evaluated using previous transcriptomics and scRNA‐seq data.^23^, ^24^ Results revealed that *BRD4* expression gradually increased in bone marrow cells from elder patients compared to younger and middle‐aged individuals (Figure 1C). Similarly, aged mice (16 M) exhibited significantly elevated *Brd4* expression in various cells, including OB and OC, compared with younger mice (1 M) (Figure 1D).

We analysed BRD4 mRNA levels in bone samples from patients with different BMDs (Table S1). As shown in Figure 1E, osteoporotic patients (RF‐BMD T‐score ≤ −2.5) had higher BRD4 mRNA levels than those with osteopenia (T‐score −1.0 to −2.5) and normal BMD (T‐score > −1.0). Inverse correlations were found between BRD4 mRNA and both RF‐BMD and LS‐BMD (Figure 1F), but not with other bone metabolic markers (Figure S1A). The lack of correlation with serum bone turnover markers may be due to the small sample size and influence of various local and global factors. To investigate Brd4's role in bone loss, we used 12‐week‐old female *Brd4^f/f^* mice, which underwent either sham or OVX surgery to induce osteoporosis. Eight weeks post‐operation, the uteri in OVX mice were significantly smaller, confirming successful osteoporosis induction (Figure S1B). Consistent with the osteoporosis phenotype,^28^ OVX mice exhibited typical osteoporotic traits, including thin trabeculae, enlarged areolae, increased fat cell density and lower BMD (Figure 1G,H). Brd4 expression was significantly higher in OVX mice compared to sham‐operated mice, as shown by immunoblotting and IHC (Figure 1I,J). scRNA‐seq data also revealed increased Brd4 expression in bone metabolism‐related cells, such as CTSK+ stromal and LYZ+ immune cells (Figure 1K,L). These findings suggest that Brd4 plays a key role in regulating trabecular bone mass and warrants further investigation in bone metabolism during aging. Uniform Manifold Approximation and Projection identified 10‐cell clusters in the bone marrow cells of osteoarthritis and osteoporosis patients (Figure 1M). Violin plot showing the elevated BRD4 expression in bone marrow cells of osteoporosis patients, compared with non‐osteoporosis patients (Figure 1N).

### 3.2 Brd4 regulates osteoclastogenesis via glycolysis

The coordination between bone formation and bone resorption is essential for maintaining bone homeostasis. Brd4 is known to play a critical role in OB differentiation and subsequent bone formation.^32^ Inhibition of Brd4 by JQ1 significantly impaired OB differentiation of iPSC‐derived MSCs, as shown by reduced Alizarin Red S staining (Figure S2A–C). Lentivirus‐mediated Brd4 depletion in OB also reduced Alizarin Red S‐positive cells and suppressed osteoblastic gene expression, including Alp, Osterix and Runx2 (Figure S2D–G).

**FIGURE 2:**
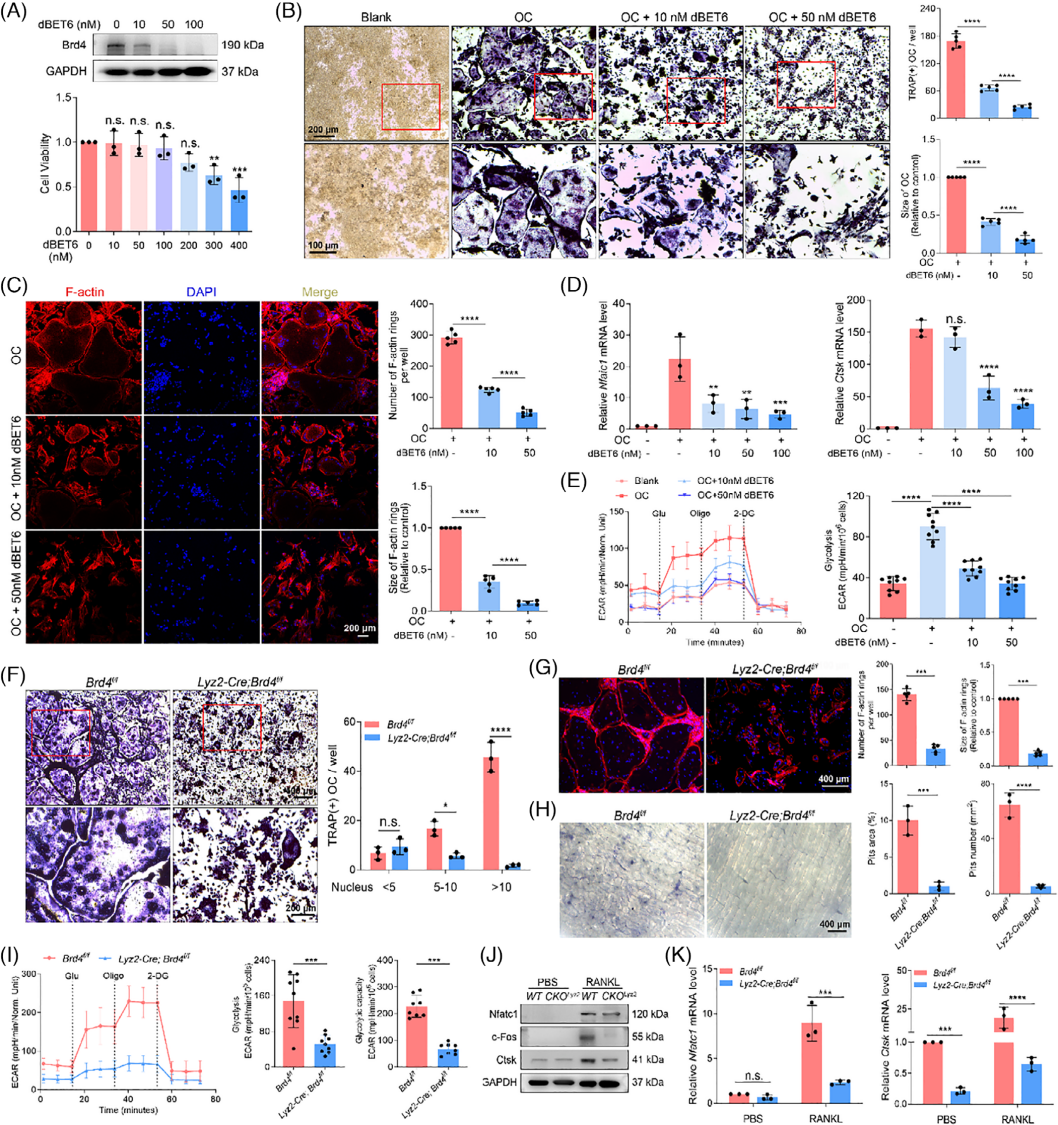
Brd4 regulates osteoclastogenesis via glycolysis. (A) Validation of Brd4 degradation by dBET6 in Raw264.7 cells with immunoblotting analysis (top). Assessment of cell viability of Raw264.7 after 24 h treatment with various concentrations of dBET6 (bottom) (*n* = 3). (B) Representative images of TRAP staining of Raw264.7 cells stimulated with RANKL and M‐CSF in the presence or absence of different concentrations of dBET6 (left), with quantitative analysis of the number and size of TRAP‐positive multinuclear cells per view (right) (*n* = 5). (C) Representative images of F‐actin ring formation of Raw264.7 stimulated with RANKL and M‐CSF in the presence or absence of dBET6 at the indicated concentrations (left), with quantitative analysis of the number and size of F‐actin rings per view (right) (*n* = 5). (D) qRT‐PCR detection of OC differentiation markers (*Nfatc1* and *Ctsk*) in the Raw264.7 cells stimulated with RANKL and M‐CSF in the presence or absence of dBET6 at the indicated concentrations (*n* = 3). (E) Seahorse analysis of extracellular acidification rate (ECAR) (left) and glycolysis (right) in the OC treated with different dBET6 concentrations for 24 h. Glu, glucose; Oligo, oligomycin; 2‐DG, 2‐deoxyglucose. (F) Representative images of TRAP staining (left) and quantification analysis (right) of BMMs from 8‐week‐old *Lyz2‐Cre; Brd4^f/f^* mice and littermate control mice after 5 days of OC induction (*n* = 3). (G) Representative images of F‐actin ring formation (left) and quantification analysis (right) of BMMs from 8‐week‐old *Lyz2‐Cre; Brd4^f/f^* mice and littermate control mice after 5 days of OC induction (*n* = 3). (H) Representative images of bone resorption pits on the bone slices stained with toluidine blue (left), with quantification of the pit area and number using Image J software (right) (*n* = 3). (I) Seahorse analysis of extracellular acidification rate (ECAR) (left) and glycolysis (right) BMMs from *Lyz2‐Cre; Brd4^f/f^* mice and littermate control mice after 5 days of OC induction. (J) Immunoblotting analysis of Nfatc1, c‐Fos and Ctsk in BMMs‐derived OC of *Brd4^f/f^* (*WT*) and *Lyz2‐Cre; Brd4^f/f^* (*cKO^Lyz2^*) mice. (K) qRT‐PCR analysis of *Nfatc1and Ctsk* mRNA expression in BMMs treated with or without RANKL (*n* = 3). Comparisons in (K) are conducted by one‐way ANOVA analyses. Comparisons in the others are conducted by Student's *t*‐test, two‐tailed. *p < .05, **p < .01, ***p < .001, ****p < .0001, n.s., not significant.

We have recently demonstrated that Brd4 is crucial for regulating macrophage polarisation and joint inflammation,^9^ yet its role in OC differentiation remains less understood. *Brd4* expression was notably up‐regulated during OC differentiation, along with the increased expression of osteoclastogenic proteins (Nfatc1, c‐Fos and Ctsk) (Figure S3A–C). To investigate whether the suppression of Brd4 by its degrader dBET6 could prevent osteoclastogenesis in Raw264.7 cells, we treated the cells with different concentrations of dBET6. Treatment with dBET6 (0–100 nM) resulted in a dose‐dependent degradation of Brd4 protein level without significant toxicity to Raw264.7 cells (Figure 2A). Brd4 degradation dose‐dependently inhibited RANKL‐stimulated osteoclastogenesis in Raw264.7 cells, as indicated by reduced TRAP and F‐actin staining (Figure 2B,C). Supporting this, treatment with dBET6 decreased the expression of *Nfatc1*, *Ctsk*, *Mmp9*, *Atp6v0d2* and *Acp5* (Figures 2D and S3D). Given that glycolysis is fundamental for OC differentiation,^4^ we then investigated whether Brd4 can also regulate OC differentiation via glycolysis. Notably, Brd4 degradation significantly reduced the ECAR associated with glycolysis during osteoclastogenesis, indicating a regulatory role for Brd4 in OC differentiation via glycolysis (Figures 2E and S3E).

Next, conditional knockout mice (*Lyz2‐cre; Brd4^f/f^)* were generated by crossing *Brd4^f/f^* with myeloid lineage‐specific *Lyz2‐Cre* mice as previously described^9^ and validated by RT‐PCR (Figure S4A–C). The knockout efficiency of Brd4 was verified by immunofluorescence (Figure S4D). BMMs from conditional knockout mice showed a significant inhibition of RANKL‐stimulated osteoclastogenesis, as demonstrated by reduced TRAP‐positive cells and impaired F‐actin ring formation (Figure 2F,G). The OC derived from BMMs of *Lyz2‐cre; Brd4^f/f^* mice also exhibited a compromised ability for bone resorption in a bone resorption assay (Figure 2H). In support of this, the loss of *Brd4* resulted in reduced glycolysis and attenuated expression of Nfatc1, c‐Fos and Ctsk proteins in BMMs during OC differentiation (Figures 2I,J and S4E). Moreover, the down‐regulation of several osteoclastogenic genes, including *Nfatc1*, *Ctsk*, *Acp5*, *Atp6v0d2*, *Mmp9*, *Dc‐stamp* and *Oc‐stamp*, was observed upon *Brd4* loss (Figures 2K, S3F,G and S4F). Collectively, these findings suggest that Brd4 is crucial for both OB differentiation and glycolysis‐mediated OC differentiation, thus playing a significant role in bone metabolism and homeostasis.

### 3.3 Brd4 depletion reverses bone loss in the pathological setting

To study Brd4's role in bone mass regulation, conditional knockout mice (*Lyz2‐cre; Brd4^f/f^*) were used. Brd4 deficiency in myeloid cells had minimal effects on body weight and limb length (Figure S5A,B). However, 14‐week‐old *Lyz2‐cre; Brd4^f/f^* mice showed significantly increased bone mass in the distal femur (Figure S5C), characterised by higher BMD, BV/TV, trabecular thickness and number, along with decreased trabecular separation (Figure S5D). No significant changes were observed in MAR, BFR/BS, serum PINP levels or Runx2 expression (Figure S5E–H), but reduced CTX levels and TRAP‐positive OCs on trabecular bone were noted (Figure S5F,I).

**FIGURE 3:**
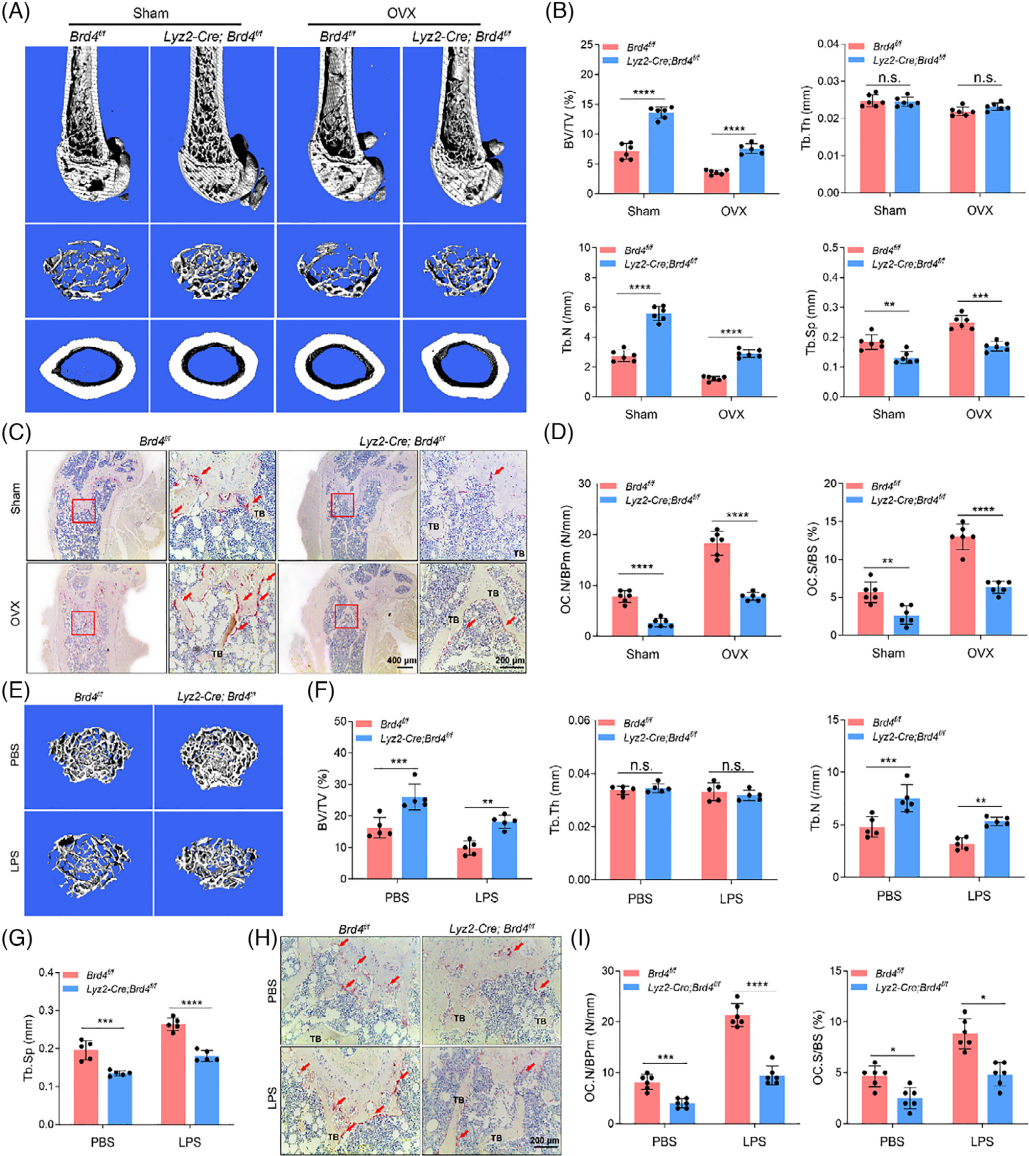
Depletion of *Brd4* in myeloid OC precursors protects mice from pathologic bone loss. (A) Representative micro‐CT images of distal femur bone of 12‐week‐old *Brd4^f/f^* and *Lyz2‐Cre; Brd4^f/f^* mice induced by sham‐operated or OVX. (B) Bone parameters (BV/TV, Tb. Th, Tb. N and Tb. Sp) of the distal femur of *Brd4^f/f^* and *Lyz2‐Cre; Brd4^f/f^* mice induced by sham‐operated or OVX as analysed by micro‐CT (*n* = 6). OVX/sham ratios were calculated for each genotype to illustrate genotype‐dependent differences. (C) Representative images of TRAP staining in the femur sections of *Brd4^f/f^* and *Lyz2‐Cre; Brd4^f/f^* mice induced by sham‐operated or OVX. The TRAP‐stained OC were denoted by the red arrow. (D) OC.N/BPm (OC number per bone parameter) and OC.S/BS (OC surface per bone surface) in C were quantified (*n* = 6). (E) Representative micro‐CT images of trabecular bone in the distal femur of 12‐week‐old *Brd4^f/f^* and *Lyz2‐Cre; Brd4^f/f^* mice treated by LPS or PBS for 8 days. (F and G) Bone parameters (BV/TV, Tb. Th, Tb. N and Tb. Sp) in the distal femur of *Brd4^f/f^* and *Lyz2‐Cre; Brd4^f/f^* mice treated by LPS or control PBS for 8 days (*n* = 5). (H) Representative images of TRAP staining in the femur sections of *Brd4^f/f^* and *Lyz2‐Cre; Brd4^f/f^* mice treated by LPS or PBS for 8 days. (I) Quantification of the number of TRAP‐positive multinuclear cells and size of OC per field (*n* = 6). All comparisons were conducted by one‐way ANOVA analyses. *p < .05, **p < .01, ***p < .001, ****p < .0001, n.s., not significant.

To further explore Brd4's role in bone loss, we applied an OVX model to mimic oestrogen‐deficiency induced osteoporosis. In *Lyz2‐Cre*; *Brd4^f/f^* mice, bone loss in the trabecular bone caused by OVX was alleviated, as evidenced by increase in BMD (63.2–65.4%), BV/TV (50.7–55.2%), Tb. Th (88.0–95.8%), Tb. N (44.4–50.7%), along with a reduction in Tb. Sp (135.2–133.0%) (Figures 3A,B and S6A,B). Although OVX led to bone loss in both control and *Lyz2‐Cre*; *Brd4^f/f^* mice, bone mass in *Lyz2‐Cre*; *Brd4^f/f^* mice was relatively preserved compared to *Brd4^f/f^* mice. TRAP staining of distal femur sections revealed that OVX significantly increased the number of TRAP‐positive OC, quantified by N.Oc/BS and Oc.S/BS. Specifically, the fold change in N.Oc/BS increased slightly from 244.1 to 263.7%, while Oc.S/BS showed a mild decrease from 237.5 to 232.4% in *Brd4^f/f^* and *Lyz2‐Cre*; *Brd4^f/f^* mice, respectively. These results suggest that Brd4 deficiency may modestly limit OC surface expansion under oestrogen‐deficient conditions (Figure 3C,D). No changes were detected in cortical bone BV/TV in the mid‐shaft of femurs of both male and female mice at 10 weeks old compared to controls (Figure S6C). Given that Brd4 is crucial for immunity regulation,^33^ its impact on inflammation‐induced bone loss was also examined using LPS‐stimulated model. LPS injection significantly decreased bone mass in the distal femur of *Brd4^f/f^* mice, however, *Lyz2‐Cre; Brd4^f/f^* mice exhibited less bone loss upon LPS administration, with substantial increase in BV/TV (60.4–69.9%), Tb. Th (93.9–97.8%), Tb. N (66.8–70.7%) and a slight increase of Tb. Sp (134.6–134.7%) (Figure 3E–G). Consistent with these observations, the loss of *Brd4* greatly decreased the presence of TRAP‐positive cells induced by LPS, as indicated by N.Oc/BS (261.2–257.9%) and Oc.S/BS (193.3–189.2.7%) (Figures 3H,I and S6D).

**FIGURE 4:**
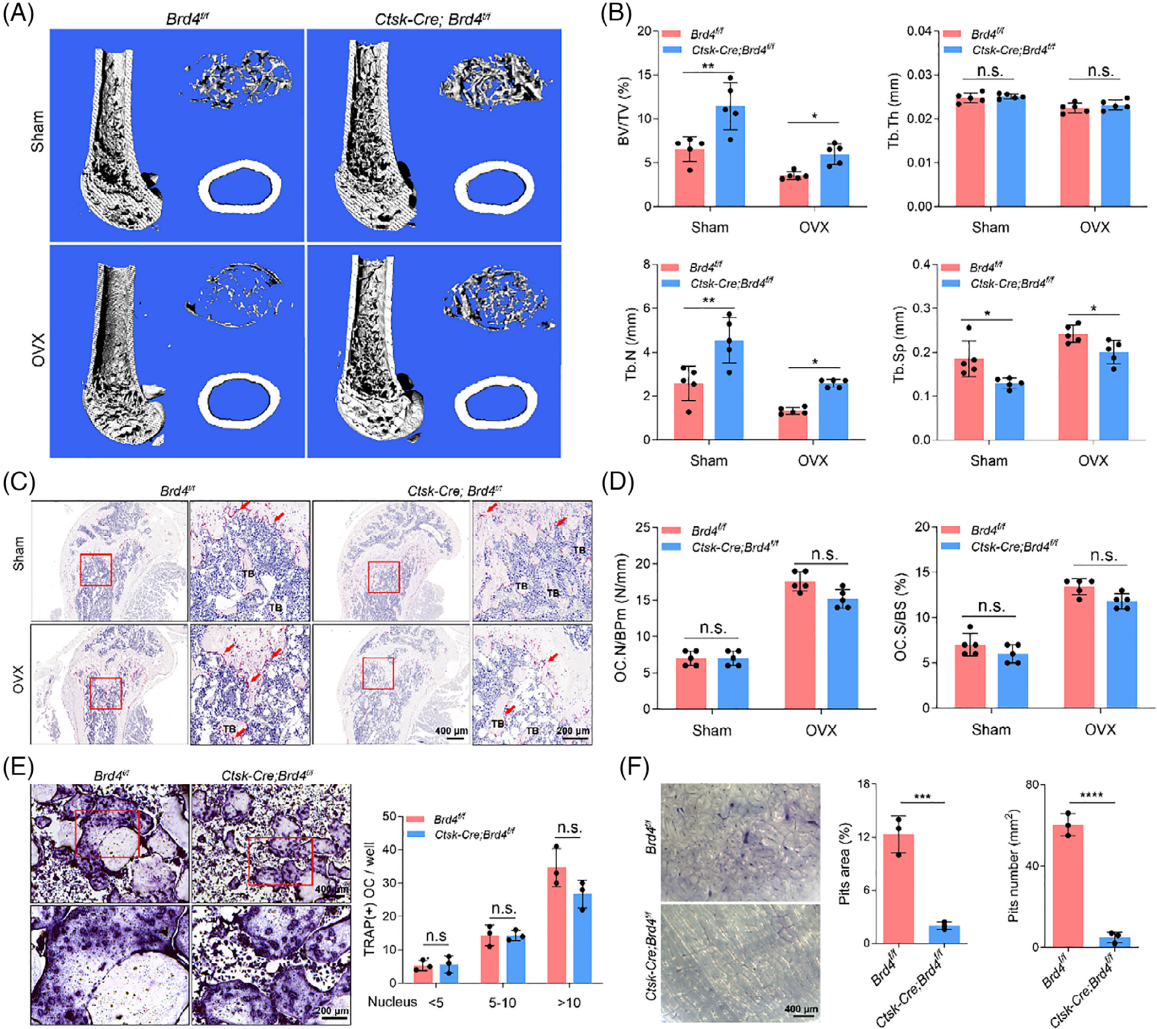
Depletion of *Brd4* in OC protects mice from pathologic bone loss by inhibiting OC activity. (A) Representative micro‐CT images of the distal femur of 12‐week‐old *Brd4^f/f^* and *Ctsk‐Cre; Brd4^f/f^* mice induced by sham‐operated or OVX. (B) Bone parameters (BV/TV, Tb. Th, Tb. N and Tb. Sp) in the distal femur of *Brd4^f/f^* and *Ctsk‐Cre; Brd4^f/f^* mice subjected to sham‐operated or OVX (*n* = 5). (C) Representative images of TRAP staining in the femur sections of *Brd4^f/f^* and *Ctsk‐Cre; Brd4^f/f^* mice induced by sham‐operated or OVX. The TRAP‐stained OC were denoted by the red arrow. (D) The TRAP‐positive OC were quantified based on OC.N/BPm and OC.S/BS (*n* = 5). (E) Representative images of TRAP staining (left) and quantification analysis (right) of BMMs from 8‐week‐old *Ctsk‐Cre; Brd4^f/f^* mice and their control littermate after 5 days of OC induction (*n* = 3). (F) Representative images of bone resorption pits on the bone slices stained with toluidine blue (left), with quantification of the pit area and number using Image J software (right) (*n* = 3). Comparisons in panels (E and F) were conducted by Student's *t*‐test, two‐tailed; in panels (B and D), by one‐way ANOVA analyses. *p < .05, **p < .01, ***p < .001, ****p < .0001, n.s., not significant.

To determine the role of Brd4 in differentiated OC during bone remodelling, *Brd4^f/f^* mice were crossed with *Ctsk‐Cre* knock‐in mice.^34^ Conditional knockout of *Brd4* in OC had a minimal effect on body weight and limb length (Figures S7 and S8A,B), but significantly increased bone mass (Figure S8C,D). This increase in bone mass is likely due to suppressed bone resorption activity, as the loss of *Brd4* in OC has minimal effect on MAR, BFR/BS, serum PINP levels, Runx2 expression and the number of OC (Figure S8E–I). Furthermore, the effect of Brd4 depletion in OC on OVX‐induced bone loss was evaluated using *Ctsk‐Cre Brd4^f/f^* mice. Consistent with results from *Lyz2‐Cre; Brd4^f/f^* mice, *Ctsk‐Cre Brd4^f/f^* mice showed increased BV/TV, Tb. Th, Tb. N and BMD and decreased Tb. Sp compared with their *Brd4^f/f^* littlemates (Figures 4A,B and S9). TRAP staining and F‐actin staining indicated that *Brd4* loss in *Ctsk‐Cre Brd4^f/f^* mice had little effect on the number and size of OC (Figure 4C–E). However, a bone resorption assay revealed a reduced number of bone resorption pits on bone slices (Figure 4F), indicating inhibited bone resorption activity. These results demonstrate that while *Brd4* depletion has a negligible effect on mature OC number and size, it significantly regulates the bone resorption activity of OC, highlighting its potential as a therapeutic target for osteoporosis.

### 3.4 Slc9b2 is a key downstream target of Brd4‐mediated OC differentiation

**FIGURE 5:**
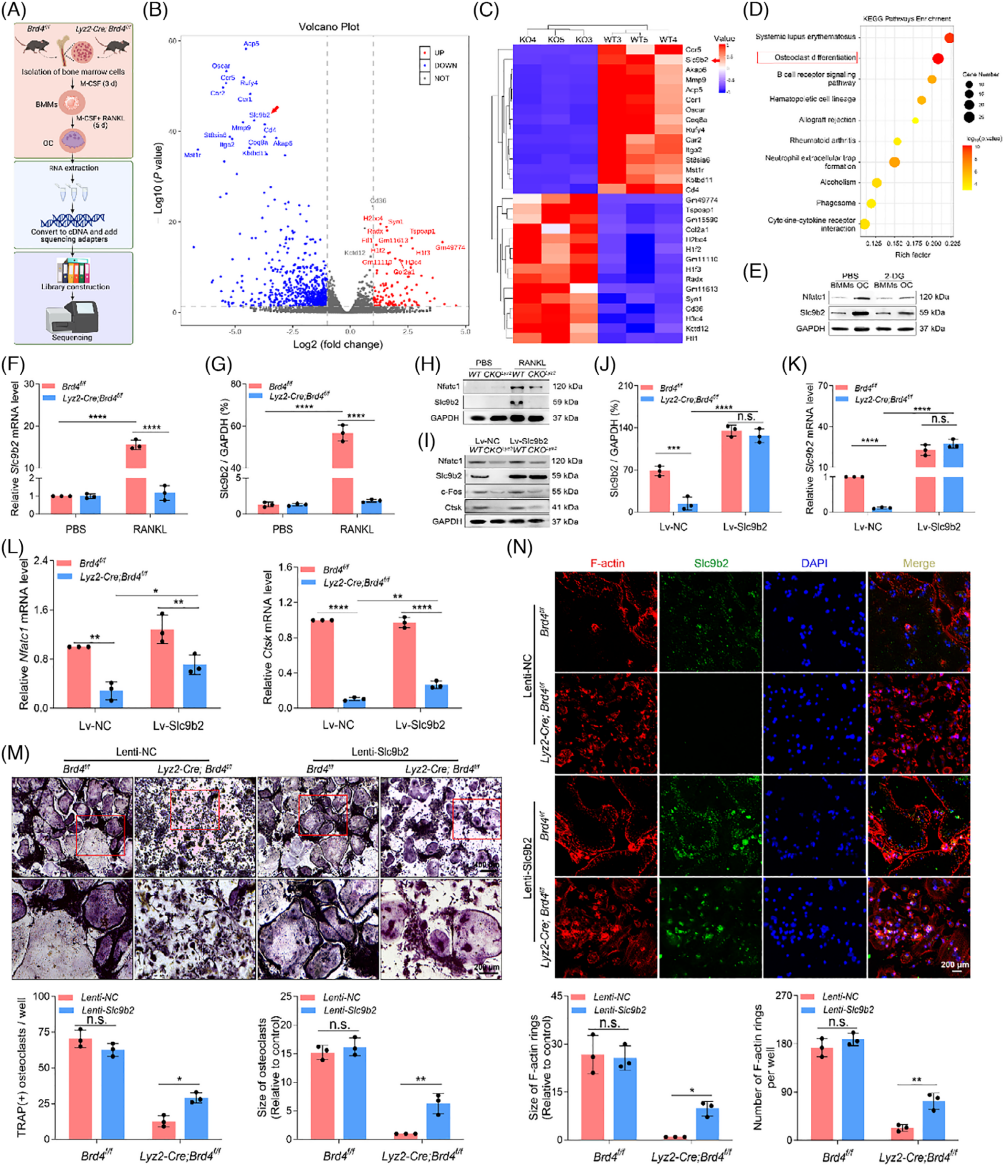
Slc9b2 is required for Brd4‐mediated osteoclastogenesis. (A) Schematic diagram illustrating the transcriptomic analysis of differentially expressed genes (DEGs) in BMMs‐derived OC of *Brd4^f/f^* and *Lyz2‐Cre; Brd4^f/f^* mice. (B) Volcano plots highlighting the up‐regulated and down‐regulated DEGs in the BMMs‐derived OC of *Lyz2‐Cre; Brd4^f/f^ group* and *Brd4^f/f^* mice. (C) Heat map showcasing the top 30 DEGs in BMM‐derived OCs, with triplicate data for each group. (D) KEGG enrichment analysis identifying the top 10 signalling pathways. (E) Immunoblotting analysis of the protein expression levels of Nfatc1 and Slc9b2 in BMMs treated with OC induction and 2‐DG or not. (F) qRT‐PCR detection of *Slc9b2* mRNA expression level in BMMs of *Brd4^f/f^* and *Lyz2‐Cre; Brd4^f/f^* mice with or without OC induction (*n* = 3). (G and H) Immunoblotting analysis of the express ion of Slc9b2 and Nfatc1 in BMMs of *Brd4^f/f^* and *Lyz2‐Cre; Brd4^f/f^* mice with OC induction or not (H), with quantitative analysis of protein expression levels of Slc9b2 G) (*n* = 3). (I and J) BMMs from *Brd4^f/f^* and *Lyz2‐Cre; Brd4^f/f^* mice with OC induction were infected with lentivirus expressing vehicle control (Lv‐NC) or Slc9b2 (Lv‐ Slc9b2) for 24 h, followed by immunoblotting to detect Slc9b2 and Nfatc1 (I), with quantitative analysis of protein expression levels of Slc9b2 (J) (*n* = 3). (K) qRT‐PCR detection of *Slc9b2* mRNA expression level in BMMs of *Brd4^f/f^* and *Lyz2‐Cre; Brd4^f/f^* mice with or without OC induction, after infected with Lv‐NC or Lv‐Slc9b2 for 24 h (*n* = 3). (L) qRT‐PCR detection of the mRNA expression levels of *Nfatc1 and Ctsk* in BMMs‐derived OC from *Brd4^f/f^* and *Lyz2‐Cre; Brd4^f/f^* mice (*n* = 3). (M) Representative images of TRAP‐stained cells in BMM‐derived OCs from *Brd4^f/f^* and *Lyz2‐Cre; Brd4^f/f^* mice infected with Lv‐NC or Lv‐Slc9b2 (top); Quantitative analysis of the number and size of TRAP‐positive multinuclear cells (bottom) (n = 3). (N) Representative images of F‐actin ring formation in BMM‐derived OCs from Lv‐NC and Lv‐Slc9b2 (top); quantitative analysis of the size and number of F‐actin rings per view (bottom) (*n* = 3). All comparisons were conducted by one‐way ANOVA analyses. *p < .05, **p < .01, ***p < .001, ****p < .0001, n.s., not significant.

To investigate the mechanism underlying the inhibitory effect of myeloid‐specific ablation of Brd4 on osteoclastogenesis, bulk RNA sequencing (RNA‐seq) was conducted to analyse transcriptomic changes. BMMs from 6 to 8 week‐old *Lyz2‐Cre*; *Brd4^f/f^* and *Brd4^f/f^* mice were induced with RANKL/M‐CSF to form OC, followed by the RNA‐seq and bioinformatic analysis (Figure 5A). A total of 700 differentially expressed genes (DEGs) were identified, with 564 down‐regulated and 136 up‐regulated, using criteria of |log_2_ (fold change)| > 1 and adjusted *p* value < .05 (Figure S10A,B). Hierarchical clustering of the top 15 down‐ and up‐regulated genes was visualised via volcano plot and heat map (Figure 5B,C). Consistent with the above in vitro and in vivo experiments, unbiased KEGG pathway analysis, performed using the list of DEGs (including both up‐regulated and down‐regulated) from the *Lyz2‐Cre*; *Brd4^f/f^* versus *Brd4^f/f^* comparison, revealed a crucial role for Brd4 in regulating OC differentiation (Figure 5D). Among the altered genes, a series of key OC‐related genes including *Ccr1*, *Ccr5* and *Kbtbd11* were suppressed in the *Lyz2‐Cre*; *Brd4^f/f^* mice (Figure S10C), validating the transcriptomic analysis. Notably, the novel sodium/hydrogen exchanger NHA2 (Slc9b2), which is up‐regulated during RANKL‐induced OC differentiation,^35^ was significantly suppressed by the inhibition of glycolysis with 2‐DG (Figures 5E and S11A) and by the loss of *Brd4* in *Lyz2‐Cre*; *Brd4^f/f^* mice compared with *Brd4^f/f^* mice upon OC induction (Figure 5F–H).

Next, we investigate whether enhanced Slc9b2 could mitigate the inhibitory effects of Brd4 ablation on OC differentiation. Overexpression of Slc9b2 was achieved by infecting BMMs with lentivirus expressing Slc9b2 (Lv‐Slc9b2), and this was confirmed by immunoblotting and mRNA analysis (Figures 5I–K and S11B). Notably, overexpression of Slc9b2 partially reversed the suppression of OC marker genes such as *Nfatc1*, *Ctsk*, *Acp5*, *Atp6v0d2*, *Dc‐stamp* and *Oc‐stamp*, induced by Brd4 knockout (Figures 5L and S11C,D). Supporting this, TRAP and F‐actin staining demonstrated that Slc9b2 overexpression restored osteoclastogenesis, which was suppressed by the loss of *Brd4* in *Lyz2‐Cre*; *Brd4^f/f^* mice (Figure 5M,N). These results collectively indicate that Slc9b2 is a novel target of *Brd4* in mediating osteoclastogenesis, suggesting a critical role of Brd4 in regulating glycolysis and OC differentiation via Slc9b2.

### 3.5 Brd4 degradation by PROTAC@PLSs reverses pathological bone loss

**FIGURE 6:**
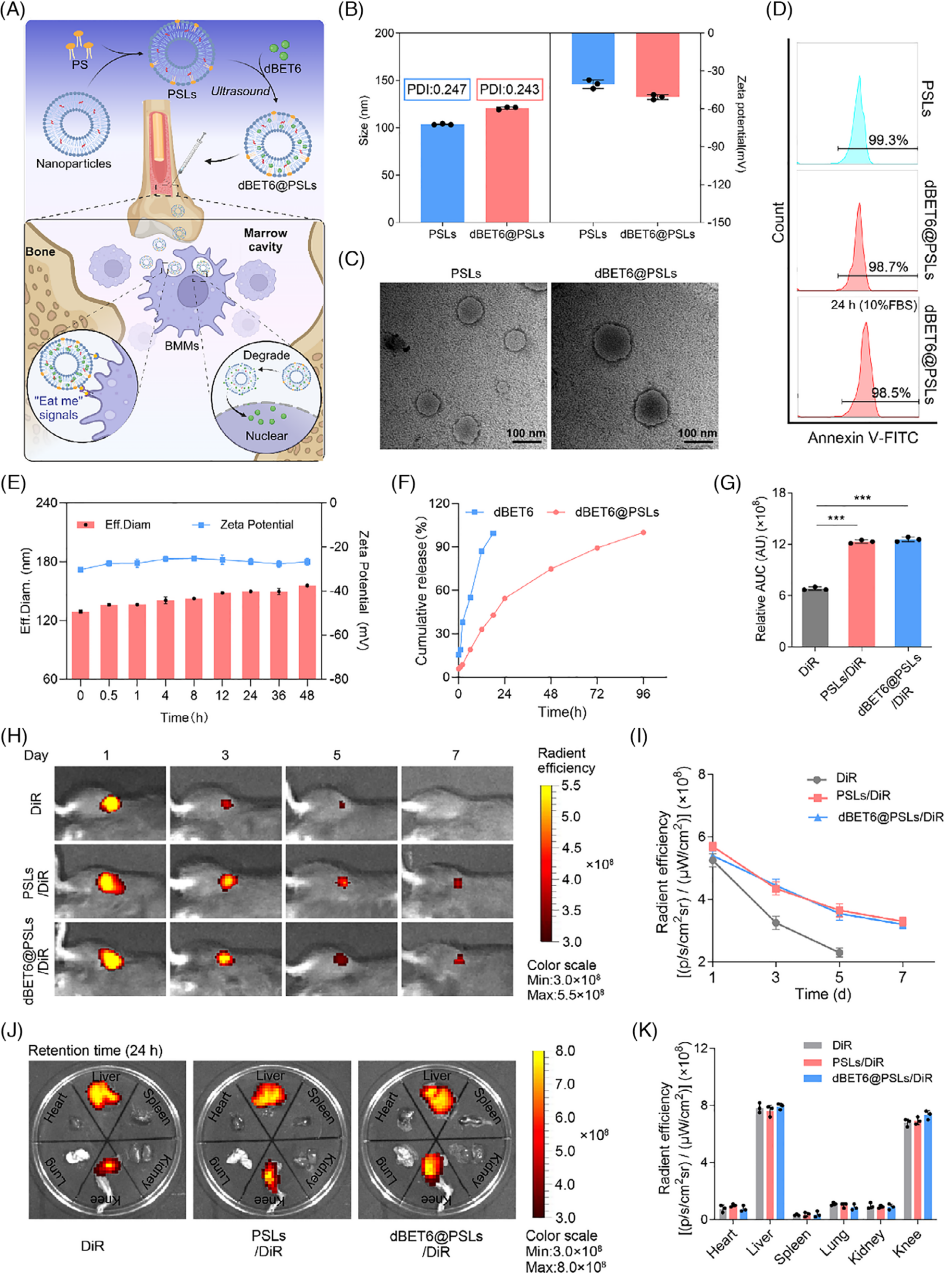
Preparation, characterisation and biodistribution of dBET6@PSLs. (A) Schematic diagram showing the composition of PSLs and dBET6@PSLs and the rationale for BMMs targeting. (B) Diameter, PDI and zeta potential of PSLs and dBET6@PSLs. (C) Representative TEM images of PSLs and dBET6@PSLs under cryogenic conditions (4°C). (D) FACS analysis of PS on the surface of PSLs and dBET6@PSLs (incubated with 10% FBS for 0 or 24 h) by Annexin V‐FITC staining. (E) Change of size, zeta potential and PDI of dBET6@PSLs incubated with 10% FBS at various times. (F) Cumulative release of free dBET6 and dBET6@PSLs in vitro. (G) Quantitative analysis of the area under the curve (AUC) using the fluorescence intensity profiles in (I) (*n* = 3). (H) Representative fluorescence images of the knee joint over 7 days post‐intramedullary injection of DiR, PSLs/DiR and dBET6@PSLs/DiR. (I) Time‐resolved quantitative analysis of radiant efficiency within femur medullary cavity following intramedullary injection of DiR, PSLs/DiR and dBET6@PSLs/DiR (*n* = 3). (J and K) Biodistribution of DiR, PSLs/DiR or dBET6@PSLs/DiR in major organs and the lower limb 24 h after intramedullary injection (*n* = 3). (J) depicts the biodistribution across major organs and the lower limb, while (K) reveals a quantitative assessment of the fluorescence intensity in various organs. Comparisons were conducted by Student's *t*‐test, two‐tailed. ***p < .001.

Recently, various methods have been developed for osteoporosis‐targeting therapeutics, such as intramedullary injection or nanoparticles modified with alendronate, peptide and preosteoclast membranes.^36^, ^37^ We have recently developed apoptotic body‐inspired PSLs to deliver the Brd4 inhibitor JQ1, specifically targeting macrophages rather than other cells.^9^ Herein, we adapted this strategy to create macrophage‐targeting PSLs loaded with the Brd4 degrader dBET6, decorated with ‘eat‐me’ signals (Figure 6A). Notably, the dBET6‐loaded PSLs (dBET6@PSLs) exhibited increased hydrodynamic diameter (103.6 ± 0.6 to 120.8 ± 1.3 nm), PDI (0.247 ± 0.012 to 0.243 ± 0.018) and zeta potential (−40.4 ± 3.4 to −50.7 ± 1.9 mV) (Figure 6B). TEM confirmed their spherical shape (∼100 nm) (Figure 6C). EE and DLE of dBET6 were 94.34 ± 0.77 and 4.87 ± 0.23%, respectively. The PS signal on the surface of PSLs and dBET6@PSLs was detected using Annexin V‐FITC,^38^ confirming that approximately 98% of the PS signal was enriched in PSLs or dBET6@PSLs (Figure 6D). Flow cytometry showed over 90% of dBET6@PSLs retained PS surface signals for 24 h (Figure 6D), maintaining stability in particle size, zeta potential and PDI in 10% FBS medium for up to 48 h (Figure 6E and Table S2). UV spectrophotometry revealed slower dBET6 release from dBET6@PSLs compared with free dBET6 (Figure 6F). In vitro uptake studies showed dose‐ and time‐dependent uptake of dBET6@PSLs/DiD by macrophages, with optimal dose (0.25 mM) and time (9 h) determined by CLSM and FACS (Figure S12). In vivo biodistribution with DiR indicated prolonged retention of dBET6@PSLs/DiR in knee joints for up to 7 days (Figure 6G–I). While dBET6@PSLs/DiR was mainly metabolised in the liver, no significant toxicity was observed in major organs(Figures 6J,K and S13).

**FIGURE 7:**
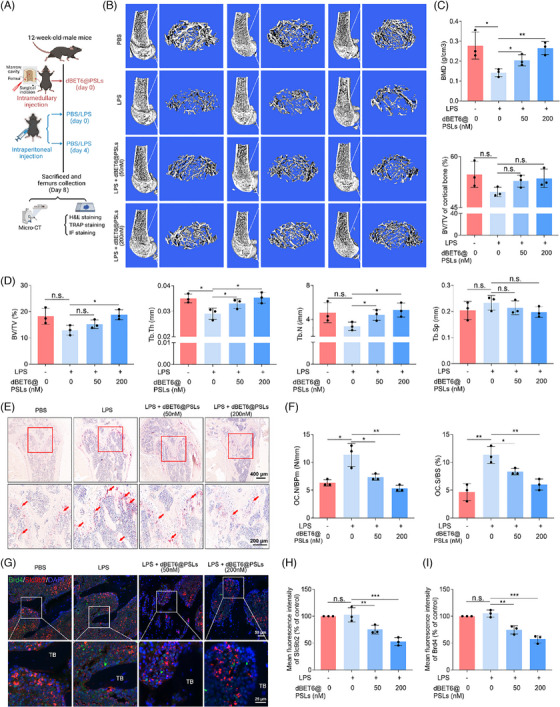
dBET6@PSLs prevents pathological bone loss via regulating Slc9b2. (A) Schematic showing the in vivo therapeutic approach administrated to 12‐week‐old WT mice. The treatment protocol involved intramedullary injections of dBET6@PSLs, followed by intraperitoneal administration of either LPS or PBS and the late pathological analysis. (B) Representative micro‐CT photographs depict the skeletal alteration in 12‐week‐old WT mice treated with 50 or 200 nM dBET6@PSLs with or without LPS induction. (C and D) Micro‐CT analysis (BMD, BV/TV of cortical, BV/TV, Tb.Th, Tb.N and Tb.Sp) of the distal femur in (B) (*n* = 3). (E) Representative images of TRAP‐stained cells in the femur sections of 12‐week‐old WT mice treated with 50 or 200 nM dBET6@PSLs with or without LPS induction. (F) The TRAP‐positive OC indicated by the red arrow were quantified with respect to OC.N/BPm and OC.S/BS (*n* = 3). (G) Representative immunofluorescence images of the distal femur stained with Brd4 (green) and Slc9b2 (red) in the 12‐week‐old WT mice treated with 50 or 200 nM dBET6@PSLs with or without LPS induction. (H, I) Quantitative analysis of Brd4 and Slc9b2 mean fluorescence intensity was performed on multiple randomly selected fields per sample (*n* = 3). All comparisons were conducted by Student's *t*‐test, two‐tailed. *p < .05, **p < .01, ***p < .001, n.s., not significant.

**FIGURE 8:**
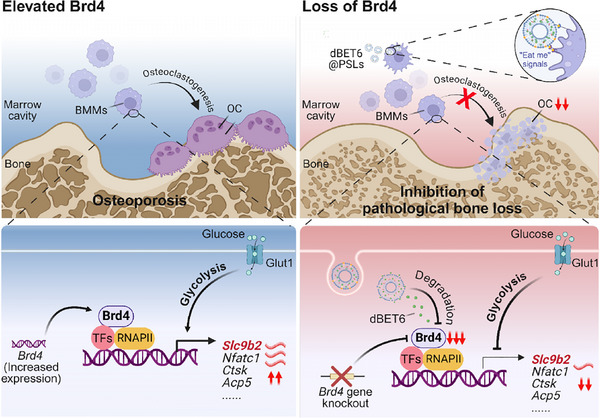
Brd4 regulates bone metabolism through Slc9b2 suppression: a targeted therapeutic approach for osteoporosis. Elevated Brd4 expression is strongly correlated with osteoporosis, primarily by promoting osteoclastogenesis. Mechanistically, Brd4 is crucial role in regulating glycolysis, a prerequisite for osteoclastogenesis (left). In contrast, the loss of Brd4 has been shown to increase basal bone mass and prevent pathological bone loss induced by OVX or LPS, through the suppression of OC markers, particularly Slc9b2. Targeting Brd4 with PROTACs loaded on PSLs (dBET6@PSLs) significantly inhibited osteoclastogenesis and alleviated pathological bone loss. These findings suggest that Brd4 inhibition could be a promising therapeutic strategy for preventing pathological bone loss, including osteoporosis.

Next, the superiority of Brd4 degradation by dBET@PSLs in vitro was verified by immunoblotting (Figure S14). For in vivo efficacy, dBET6@PLSs were intramedullary injected into the distal femur, followed by intraperitoneal administration of either LPS or PBS, and subsequently subjected to histological analysis (Figure 7A). Severe trabecular bone loss was observed in the LPS‐treated mice, as shown by micro‐CT analysis and H&E staining. These results indicate that Brd4 degradation by dBET6 tended to attenuate LPS‐induced bone loss, as evidenced by consistent trends of increased BMD and trabecular thickness. Although differences in BV/TV, Tb.N and Tb.Sp did not reach statistical significance, the histological observations and micro‐CT data together support this potential effect (Figures 7B–D and S15). TRAP staining further revealed reduced TRAP‐positive OC in LPS‐induced acute bone loss models treated with dBET6@PLSs (Figure 7E,F). These results validate the strong antiosteoporotic effects of the dBET6@PLSs. In accordance with the above findings, loss of Brd4 induced by the dBET6@PLSs also suppressed the expression of Slc9b2, as demonstrated by the co‐staining with Brd4 (Figure 7G–I). These results together suggest that Brd4 targeting could alleviate osteoporosis progression by modulating Slc9b2 expression in OC, highlighting Brd4 as a promising target for pathological bone loss treatment (Figure 8).

## 4 DISCUSSION

Understanding the role of epigenetic regulators in osteoclastogenesis could open up new avenues for treating bone‐related conditions like osteoporosis. Recent studies have highlighted the versatile role of Brd4 not only in osteogenesis and adipogenesis but also in chondrogenesis, where it regulates the expression of critical transcription factors such as Runx2 and Sox9, respectively.^32^, ^39^ However, the role of Brd4 in osteoporosis in vivo remains unclear. In the present study, Brd4 expression levels were found to be elevated in bone specimens from osteoporotic patients and in pathological models of osteoporosis in mice. Additionally, we observed that the expression of Brd4 was elevated in aging samples from both human and mice, and it would be valuable to investigate its role in either senile or OVX‐induced osteoporosis in the near future.

Several lines of evidence support that Brd4 functions as a master regulator of bone metabolism, primarily through the regulation of osteoclastogenesis. First, a strong correlation between Brd4 expression levels with BMD was observed in both human and mice specimens (Figure 1), despite the presence of co‐pathologies associated with osteoarthritis and osteoporosis. Second, by applying two types of Brd4 conditional knockout mice, we demonstrated that Brd4 deletion in *Lyz2‐Cre; Brd4^f/f^* mice markedly impaired osteoclast formation and bone resorption, whereas *Ctsk‐Cre; Brd4^f/f^* mice maintained OC morphology but exhibited reduced resorptive activity (Figures 2, 3, 4 and 7), highlighting the stage‐specific role of Brd4 in osteoclastogenesis and bone metabolism. Although Ctsk‐Cre mice have been reported to cause unintended germline deletions,^40^ In our experiments, we minimised the risk of germline recombination by using male Ctsk‐Cre carriers and genotyping offspring, and the resulting phenotypes were consistent with OC‐specific effects. Importantly, parallel validation with *Lyz2‐Cre; Brd4^f/f^* mice, which showed similar defects in OC differentiation and Slc9b2 down‐regulation, confirmed that Brd4 regulates OC function through Slc9b2 independent of the Cre driver. Third, OC largely differentiated from macrophages through the enhancement of glycolysis,^4^ herein, we also found that the loss of *Brd4* compromised OC differentiation by suppressing glycolysis and that therapeutic targeting of Brd4 effectively alleviated pathological bone loss (Figures 2 and 7).

Although *Brd4* is reported to regulate *c‐Myc*, a key transcription factor in OC metabolism and differentiation,^41^ we observed no significant changes in c‐Myc mRNA levels in myeloid‐specific *Brd4*‐deficient OCs. This suggests that *Brd4*’s inhibitory effects on osteoclastogenesis in our study are unlikely mediated via *c‐Myc*, but rather through alternative transcriptional programs. To further elucidate the underlying mechanism, we conducted transcriptomic sequencing and bioinformatics analysis and identified Slc9b2 as a target gene (Figure 5). Slc9b2, also known as NHA2, encodes proteins that belong to the solute carrier family as ion transporters.^42^ Dysregulation of Slc9b2 has been reported to contribute to various diseases, including hypertension,^43^ kidney disease,^44^ and disorders of the nervous system. However, the specific function of Slc9b2 in osteoporosis is not as well‐characterised as other family members. Recent studies have shown that Slc9b2 is strongly up‐regulated during RANKL‐induced OC differentiation and is selectively expressed in OC.^35^ Inhibition or silencing of Slc9b2 suppresses the formation of large multinuclear OC and the bone resorption process.^45^ On the other hand, studies of Slc9b2‐deficient mice indicate that Slc9b2 is dispensable for OC differentiation and bone resorption both in vivo and in vitro.^46^ In our study, we similarly found that although Slc9b2 is dispensable for OC differentiation and bone resorption, yet it plays a crucial role in the regulation of osteoclastogenesis mediated by Brd4. The differing outcomes between studies may be attributed to variations in experimental context and methodology, such as the use of global versus conditional knockout mice, differing conditions for OC induction and variations in gene knockout efficiency. Therefore, further investigation into the role of Slc9b2 in OC and osteoporosis is essential. In addition, further research aimed at delineating the regulatory role of Brd4 in glycolysis and the expression of Slc9b2 could provide deeper insights into the mechanisms of bone metabolism.

Recently, various nanocarriers, including polymers, lipid nanoparticles, exosomes and synthetic nanoparticles, are employed in osteoporosis treatment to improve the delivery and efficacy of therapeutic agents.^47^ These strategies involve the use of drug‐carrying nanoparticles that bind specifically to bone tissue, facilitating their aggregation and maximising their therapeutic impact.^19^ Moreover, the surface of these nanoparticles can be tailored with cell/tissue‐targeting molecules like bisphosphonates or OC/OB‐targeting peptides, thereby enhancing the drug's distribution within bone tissue.^18^, ^48^ We have recently developed ‘eat‐me’ signal‐inspired PSLs to enhance macrophage targeting specificity in a series of inflammatory diseases (Figure 6).^9^, ^49^ In this study, similar PSLs loaded with Brd4 PROTACs (dBET6) were developed and found to effectively alleviated pathological bone loss, including osteoporosis, primarily through the suppression of osteoclastogenesis. Although some micro‐CT parameters, such as BV/TV, Tb.N and Tb.Sp, did not reach statistical significance, we attribute this to the relatively small sample size (*n* = 3) and potential off‐target effects of the compound. Nonetheless, the consistent trends observed across multiple bone parameters support the therapeutic potential of dBET6 in bone loss disorders (Figures 7 and 8). Current drugs for inhibiting bone resorption include bisphosphonates, denosumab, calcitonin and oestrogen therapy. While effective in reducing bone loss and fracture risk, these treatments can have side effects, including gastrointestinal issues, blood clots, jaw osteonecrosis and rebound bone loss. Long‐term use also carries risks such as atypical fractures.^50^ PROTACs differ from traditional inhibitors by utilising the ubiquitin–proteasome system to degrade target proteins, thus overcoming issues like drug resistance caused by protein overexpression or mutations. Unlike conventional drugs that require an active site for binding, PROTACs can target previously ‘undruggable’ proteins. They offer a catalytic mechanism, enabling sustained protein degradation with lower doses and potentially longer‐lasting therapeutic effects. Given these advantages, several PROTACs (e.g., ARV‐110 and ARV‐47) have shown promising early‐stage clinical trial results for treating prostate and breast cancer.^51^ Therefore, dBET6@PSLs were successfully constructed to improve their short half‐life in circulation and enhance retention at the disease site.

## 5 CONCLUSION

Our results show that Brd4 is elevated in osteoporotic patients and mice. Brd4 deficiency increases bone mass in both sham‐operated and OVX mice. We also demonstrate that Brd4 depletion inhibits OB and OC differentiation, with a stronger effect on OC differentiation. Specifically, Brd4 loss in bone marrow monocytes increases basal bone mass and prevents OVX‐ and LPS‐induced bone loss. Ubias screening revealed that Brd4 loss affects Slc9b2 expression via glycolysis, and targeting Brd4 in macrophages alleviates pathological bone loss by suppressing Slc9b2 expression. Given that inhibition of OC activation in subchondral bone suppresses type‐H vessels and decreases oxygen concentration,^52^ and that Brd4 is crucial in inflammatory regulation,^9^ it is presumed that depletion of Brd4 might simultaneously alleviate degenerative diseases including osteoporosis and osteoarthritis.

## AUTHOR CONTRIBUTIONS

*Data curation, investigation, visualisation and writing—original draft*: Xiaohe Wang and Fangji Luo. *Data curation, investigation and visualisation*: Guiqiang Miao, Boyuan Zheng and Chenhao Xu. *Providing technical help*: Vincent Kam Wai Wong, Yuanshu Peng and Rong Zeng. *Writing—review and editing*: Jinzhu Pang, Xuguang Zhang and Zhenyu Ju. *Supervision*: Zhengang Zha, Xiaogang Wang and Xiaofei Zheng. *Conceptualisation, resources, supervision, writing—original draft, writing—review and editing*: Huan‐Tian Zhang.

## CONFLICT OF INTEREST STATEMENT

The authors declare no conflicts of interest.

## ETHICS STATEMENT

All experimental procedures received approval from the Ethics Committee of the First Affiliated Hospital of Jinan University (Ethical approval number: KY‐2021‐065). All participants were informed about the study's purpose and provided written informed consent prior to surgery. All procedures involving animals were approved by the Institutional Animal Care and Use Committee of Jinan University (Ethical approval number: IACUC‐20230102‐05) and adhered to the guidelines outlined in the ‘Guide for the Care and Use of Laboratory Animals’ published by the National Institute of Health in China.

## Supporting information



Supporting Information

Supporting Information

## Data Availability

The data supporting the findings of this study are available from the corresponding author upon reasonable request.
