# Supplementary material for: Loss of Brd4 alleviates pathological bone loss via Slc9b2 suppression in osteoclastogenesis
Source: Clin Transl Med. 2025 Oct 20;15(10):e70496. doi: 10.1002/ctm2.70496 (PMC12536888; doi:10.1002/ctm2.70496)
Supplement: Supplementary file 1 — Supporting Information [file CTM2-15-e70496-s002.docx]

**Supplementary Information**

**Loss of Brd4 Alleviates Pathological Bone Loss via Slc9b2 Suppression in** **Osteoclastogenesis**

**This PDF file includes:**

Supplementary Text: Experimental Section

Figure S1 to S15

Figure S1. Correlation between Brd4 expression and osteoporosis status

Figure S2. Effect of *Brd4* deficiency on the osteogenic differentiation of indicated cells

Figure S3. Brd4 regulates osteoclastogenesis via glycolysis

Figure S4. Conditional knockout of *Brd4* suppresses osteoclastogenesis

Figure S5. Loss of *Brd4* in OC progenitors has a marginal effect on bone formation

Figure S6. Depletion of *Brd4* in OC progenitors protects mice from pathological bone loss

Figure S7. Establishment and validation of OC *Brd4* conditional knockout mice

Figure S8. Deletion of *Brd4* in OC leads to high bone mass

Figure S9. Loss of *Brd4* in OC protects mice from pathological bone loss

Figure S10. Identification of Brd4 target genes using RNA-seq

Figure S11. Overexpression of Slc9b2 partially rescues the Brd4-suppressed OC differentiation

Figure S12. Cellular uptake of dBET6@PSLs/DiD by BMMs

Figure S13. Toxicity of dBET6@PSLs in mice

Figure S14. The degradation efficiency of BRD4 by dBET6@PSLs *in vitro*

Figure S15. dBET6@PSLs reduces fat cell density

Tables S1 to S4

Table S1. A detailed information of each patient enrolled for analysis

Table S2. Change of size, zeta potential, and PDI of dBET6@PSLs incubated with 10% FBS at various times

Table S3. Primers used for genotyping of BRD4 conditional knockout mice

Table S4. The primers used for quantitative RT-PCR

References (1-2)

**Supplementary Text**

**Experimental Section**:

*Histomorphometry analysis for bone formation*

For dynamic histomorphometric analysis, mice received calcein (Sigma, C0875) injection at a dose of 20 mg/kg body weight for seven days, with an additional dose administered one day prior to euthanasia. The mineral apposition rate (MAR) and bone formation rate/bone surface (BFR/BS) were assessed using a laser scanning confocal microscope (Zeiss LSM 880, Germany).

*Whole-Mount Skeletal staining*

The staining was performed as we described previously [1]. Briefly, after the mice were eviscerated and the skin excised, the samples were immersed in acetone for 48 h following an overnight fixation in 95% ethanol. The skeletons were then stained in Alcian blue and Alizarin Red S solution (Beyotime, Cat#C0148S, China) for 3 d at 37℃, followed by sequential clearance in 1% potassium hydroxide (KOH). The skeletons were then immersed in 1% KOH/20% glycerol for 3 d and subjected to increasing concentrations of glycerol/ethanol solution (20%, 50%, and 100%) for 1 d each. Finally, the stained skeletons were dehydrated in glycerol for imaging and subsequent storage.

*Serum Assays*

To assess the bone turnover biomarkers, serum samples were collected from all mice. The concentrations of soluble receptor activator of carboxy-terminal telopeptides of type I collagen (CTX-1), a serum bone-resorption marker, and procollagen I N-terminal propeptide (PINP), a serum bone-formation marker, in the blood plasma were quantified using Enzyme-linked immunosorbent assay (ELISA) kits as previously described.[1]

*Cellular uptake assay*

The uptake efficiency of dBET6@PSLs by bone marrow macrophages (BMMs) was evaluated using confocal laser scanning microscopy and quantified via FACS.[2] dBET6@PSLs were first labeled with DiD dye, then varying concentrations of dBET6@PSLs/DiD (8.41, 42.07, 84.14, and 168.27 ng/mL) were added to the BMMs seeded into confocal dishes for different durations (3, 6, 9, and 12 h). After incubation, the cells were fixed with 4% PFA and permeabilization with 0.1% Triton X-100. The cellular nucleus and cytoskeleton were stained with DAPI and Actin-Tracker Green-488 (Beyotime, C2201S, China), respectively, for subsequent quantitative analysis.

*Osteogenic induction and Alizarin Red S staining*

For osteogenic induction, iPSC-MSCs were plated at a density of 5×10^5^ cells per well in 6-well plates or 1.2×10^5^ cells per well in 24-well plates. Following treatment with various concentrations of JQ1 (100~1000 nM) for 24 h, iPSC-MSCs were cultured in osteogenic medium supplemented with 10% FBS, 50 μg/mL ascorbate, 10 μmol/L β-glycerophosphate, 0.1 μmol/L dexamethasone, and 10 μmol/L glutamine. The medium was refreshed every three days. Calcified nodules were stained with 0.1% Alizarin Red S solution for 30 min, then the results were observed and quantified as previously described. [1]

*Genotype identification*

The genotype of the newborn mice was determined using the Quick Genotyping Assay Kit for Mouse Tail (Beyotime, D7283M, Shanghai, China) following the manufacturer's protocol. A small tail tissue sample was collected, and DNA was extracted. PCR was then used to amplify specific genetic markers. The amplified DNA fragments were separated and visualized through gel electrophoresis, and the resulting band patterns were analyzed to identify the genotype.


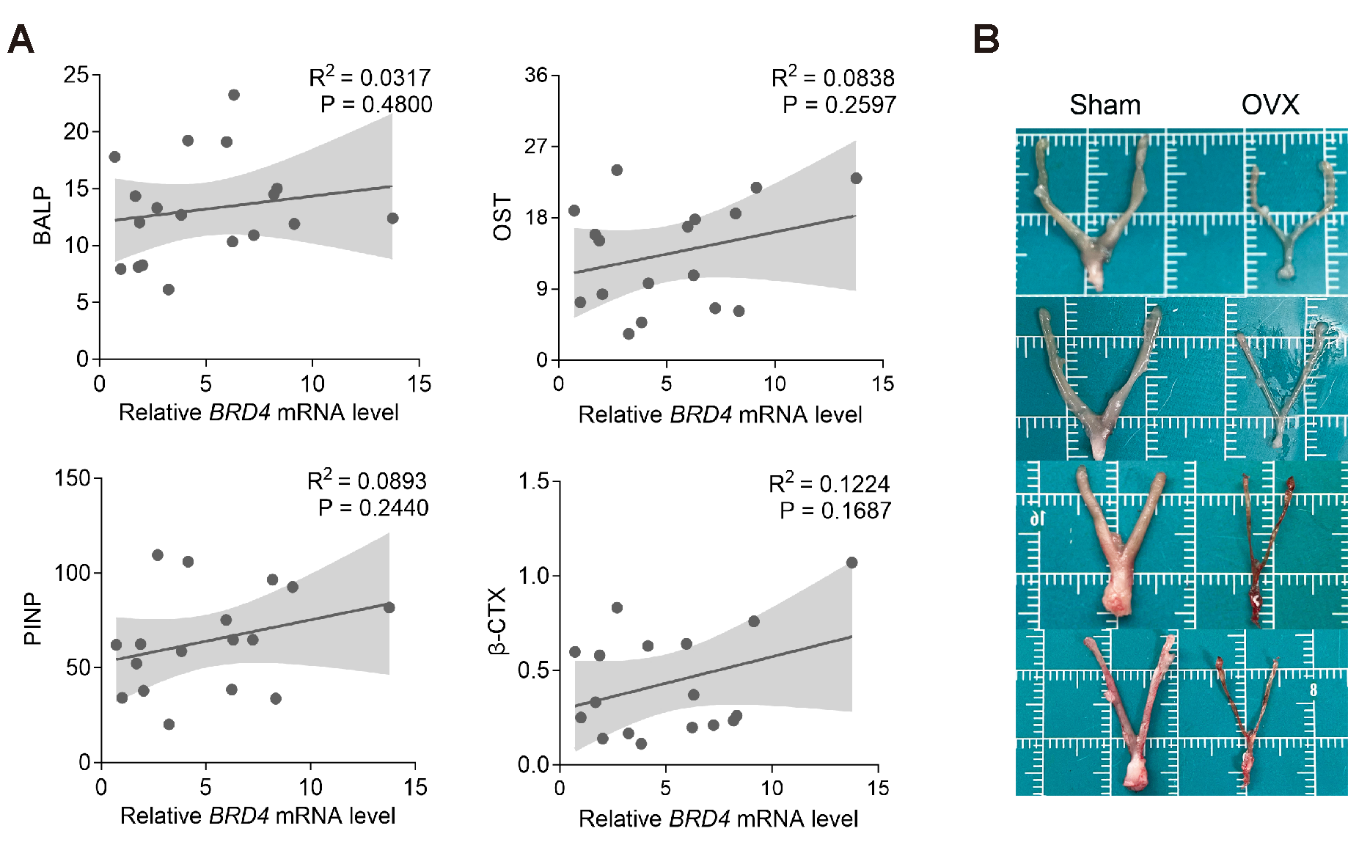


**Figure S1** **Correlat****ion between Brd4 expression and osteoporosis status.** **A** Correlation analysis between the mRNA expression of *Brd4* and bone metabolism markers (bone alkaline phosphatase (BALP), amino-terminal propeptide of type I collagen (PINP), osteocalcin (OST), and C-terminal telopeptide of type I collagen (β-CTX) in patients with varying bone mineral densities (BMD). **B** Images of uterine in mice with sham-operated or ovariectomy (OVX) surgery after 8 weeks. Comparisons were conducted using simple linear regression.


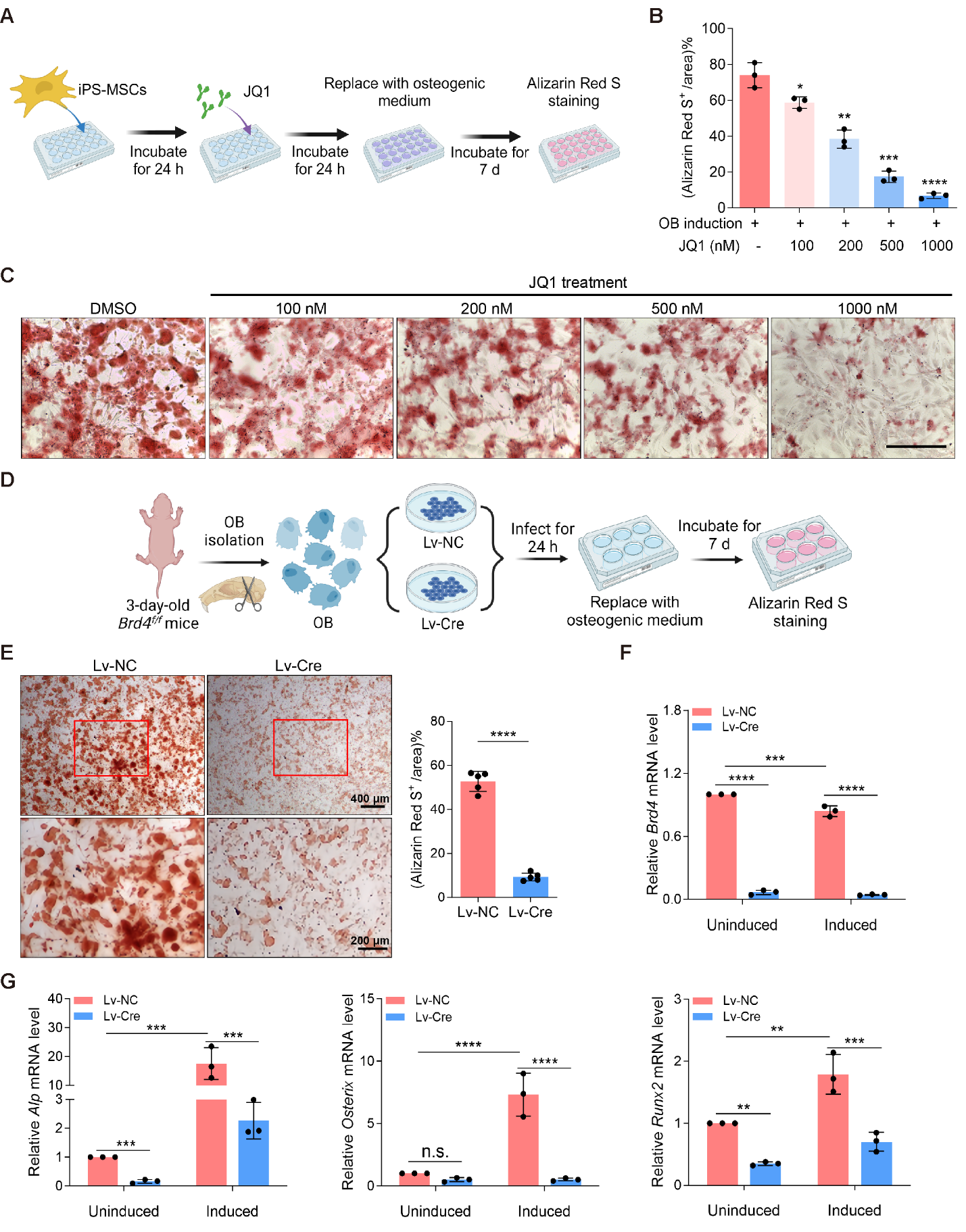


**Figure S2** **Effect of *Brd4* deficiency on the osteogenic differentiation of indicated cells.** **A** Schematic diagram illustrating the osteogenic differentiation protocol for iPSC-MSCs and the treatment with JQ1. **B, C** Representative images and the quantitative analysis of Alizarin Red S staining of iPSC-MSCs treated with indicated concentrations of JQ1 after osteogenic induction (n = 3). **D** Schematic diagram illustrating the isolation and treatment of OB. **E** Representative images of Alizarin Red S staining of OB infected with control lentivirus (Lv-NC) or lentivirus expressing Cre (Lv-Cre) (left). Quantitative analysis of the percentage of Alizarin Red S-stained area (right) (n = 5). **F, G** qRT-PCR analysis of the mRNA level of *Brd4* (F) and the osteogenic biomarker genes (*Alp*, *Osterix*, and *Runx2*) (G) in the OB infected with Lv-NC or Lv-Cre with or without osteogenic induction (n = 3). Comparisons in B, E) were conducted by Student’s t test, two-tailed; in F, G), by one-way ANOVA analyses. **p* < 0.05, ***p* < 0.01, ****p* < 0.001, *****p* < 0.0001, n.s., not significant.


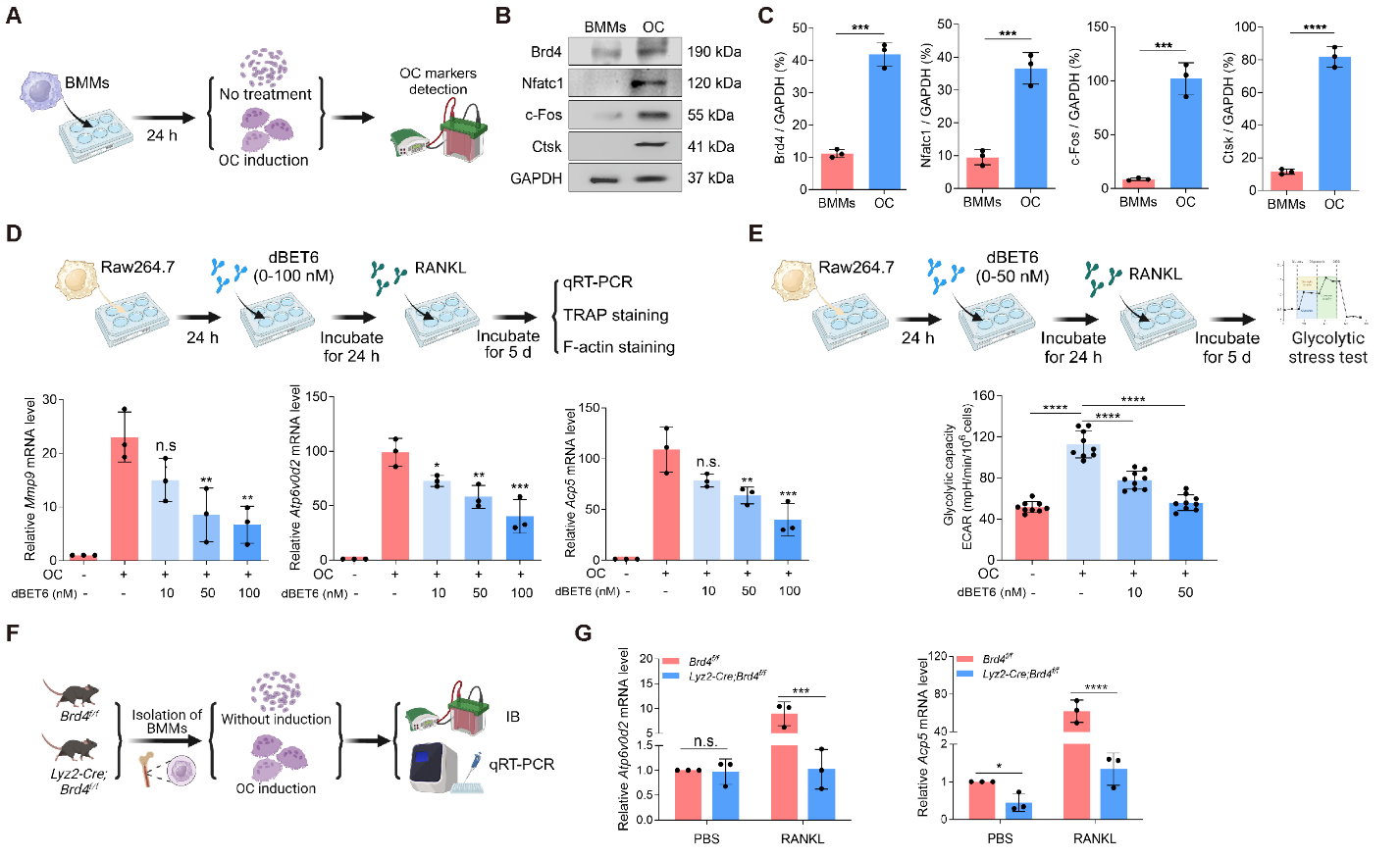


**Figure S3 Brd4 regulates osteoclastogenesis via glycolysis.** **A** Schematic diagram illustrating the changes in protein expression levels of OC markers in BMMs with or without OC induction. **B and C** Brd4 protein expression in BMMs was measured by immunoblotting with or without OC induction (n = 3). **D** qRT-PCR detection of *Mmp9*, *Acp5*, and *Atp6v0d2* in Raw264.7 cells stimulated with RANKL in the presence or absence of dBET6 at indicated concentrations (n = 3). **E** Seahorse analysis of glycolytic capacity in the OC treated with diﬀerent dBET6 concentrations for 24 h. **F** Schematic diagram illustrating the changes in protein and mRNA expression levels of OC markers in BMMs-derived OC of *Brd4^f/f^* and *Lyz2-Cre; Brd4^f/f^* mice with or without OC induction. **G** qRT-PCR analysis of *Atp6v0d2 and Acp5* mRNA expression in BMMs treated with or without RANKL (n = 3). Comparisons in C, D, E were conducted by Student’s t-test, two-tailed; In G), by one-way ANOVA analyses.. **p* < 0.05, ***p* < 0.01, ****p* < 0.001, *****p* < 0.0001, n.s., not significant.


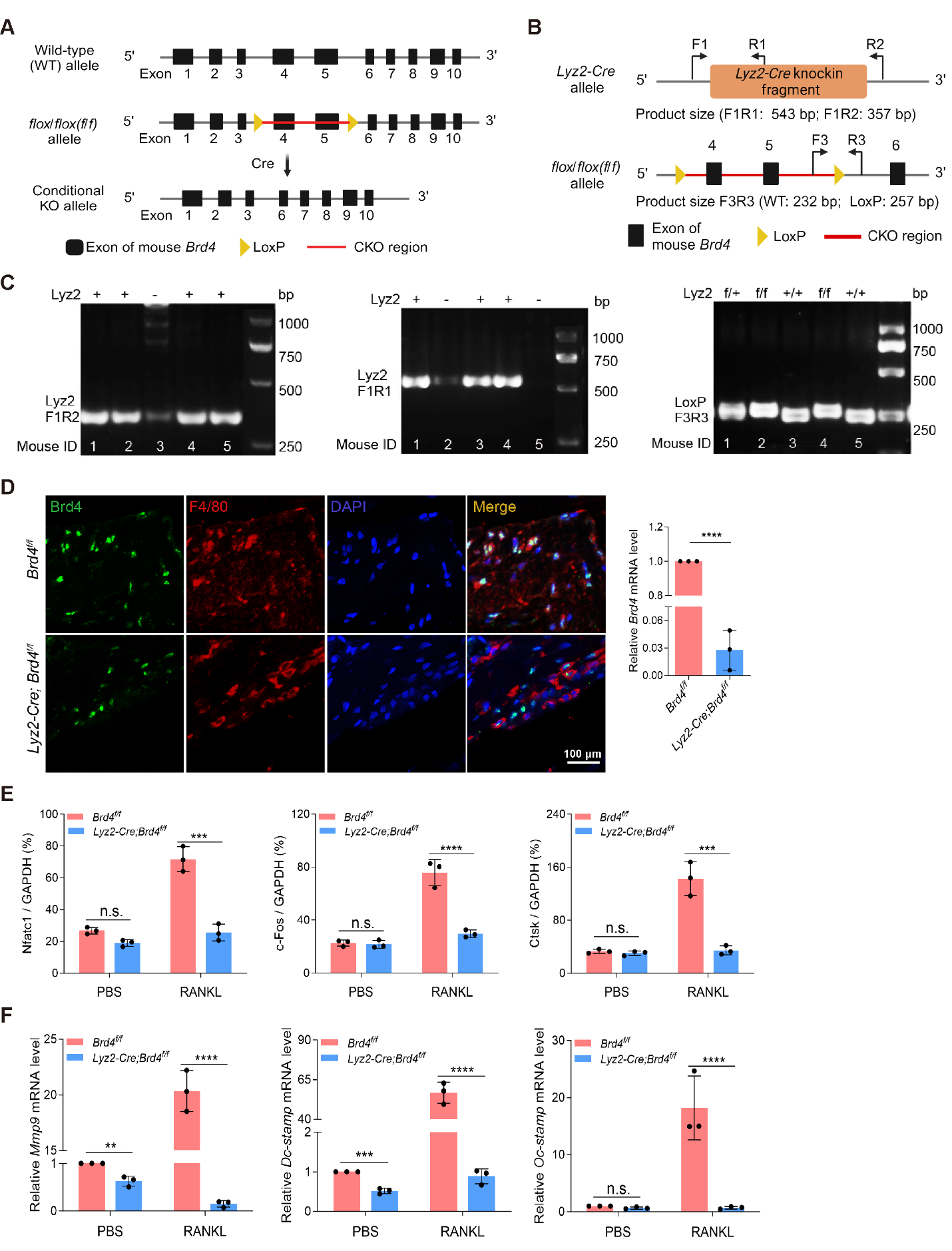


**Figure S4** **Conditional knockout of *Brd4* suppresses osteoclastogenesis.** **A** Schematic representation of construction of *Brd4* conditional knockout (*Brd4^f/f^*) mice. **B** Genotyping of *Lyz2-Cre; Brd4^f/f^* mice and their control littermates by RT-PCR. **C** *Brd4^f/f^* and *Lyz2-Cre; Brd4^f/f^* mice were genetically validated via RT-PCR genotyping. The genotypes of the five mice are *Lyz2^+/+^**; Brd4**^f/+^, Lyz2^-/+^; Brd4^f/f^, Lyz2^+/-^; Brd4**^+/+^, Lyz2^+/+^; Brd4^f/f^, and Lyz2^-/+^; Brd4^+/+^*, respectively. **D** Knockout efficiency of *Brd4* in BMMs was evaluated via immunofluorescence (left) and qRT-PCR (right) (n = 3). **E** Relative quantification of the protein expression levels of Nfatc1, c-Fos and Ctsk in BMMs-derived OC of *Brd4^f/f^* (*WT*) and *Lyz2-Cre; Brd4^f/f^* (*cKO^Lyz2^*) mice (n = 3). **F** qRT-PCR detection of the mRNA level of *Mmp9*, *Dc-stamp*, and *Oc-stamp* in BMMs treated with or without RANKL. Comparisons in D) were conducted by Student’s t test, two-tailed; In E, F), by one-way ANOVA analyses. ***p* < 0.01, ****p* < 0.001, *****p* < 0.0001, n.s., not significant.


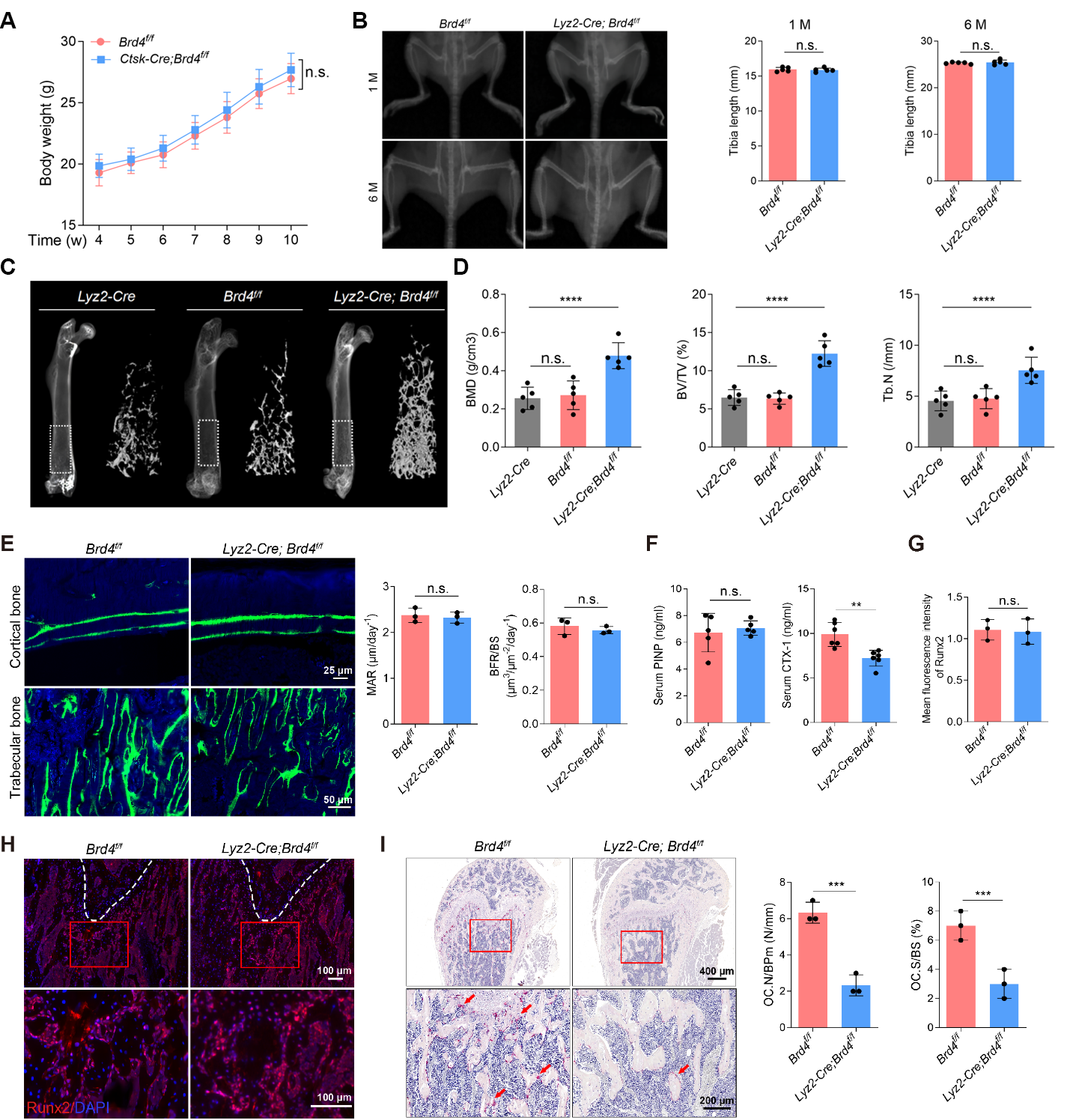


**Figure S5** **Loss of *Brd4* in OC progenitors has a marginal effect on bone formation.** **A** Growth curve showing the body weight gained between *Brd4^f/f^* and *Lyz2-Cre; Brd4^f/f^* mice from 4 to 10 weeks of age. **B** X-ray images of *Brd4^f/f^* and *Lyz2-Cre; Brd4^f/f^* mice at 1 and 6 months old (left). Quantitative analysis of the tibia length of mice at different ages (right) (n = 5). **C** Representative micro-CT images of the femur of 12-week-old mice with different genotypes. **D** Quantitative measurements of BMD, BV/TV, and Tb.N (n = 5). **E** Visualization of double calcein labeling in *Brd4^f/f^* and *Lyz2-Cre; Brd4^f/f^* mice at 4 weeks of age (left), along with quantification of the MAR and BFR/BS (right) (n = 3). **F** ELISA detection of PINP (serum bone-formation marker) and CTX-1 (serum bone-resorption marker) in serum of 8-week-old mice (n = 5). **G, H** Representative immunofluorescence images (H) and quantitative analysis of Runx2 (red) (G) in the distal femur sections of *Brd4^f/f^* and *Lyz2-Cre; Brd4^f/f^* mice at 8 weeks of age (n = 3). White dashed lines outline the growth plate of the distal femur. **I** Representative images of TRAP staining in the distal femur of 8-week-old *Lyz2-Cre; Brd4^f/f^* mice and control littermates (left), with the quantification of OC.N/BPm and OC.S/BS (right) (n = 3). Comparisons were conducted by Student’s t test, two-tailed. **p* < 0.05, ***p* < 0.01, ****p* < 0.001, *****p* < 0.0001, n.s., not significant.


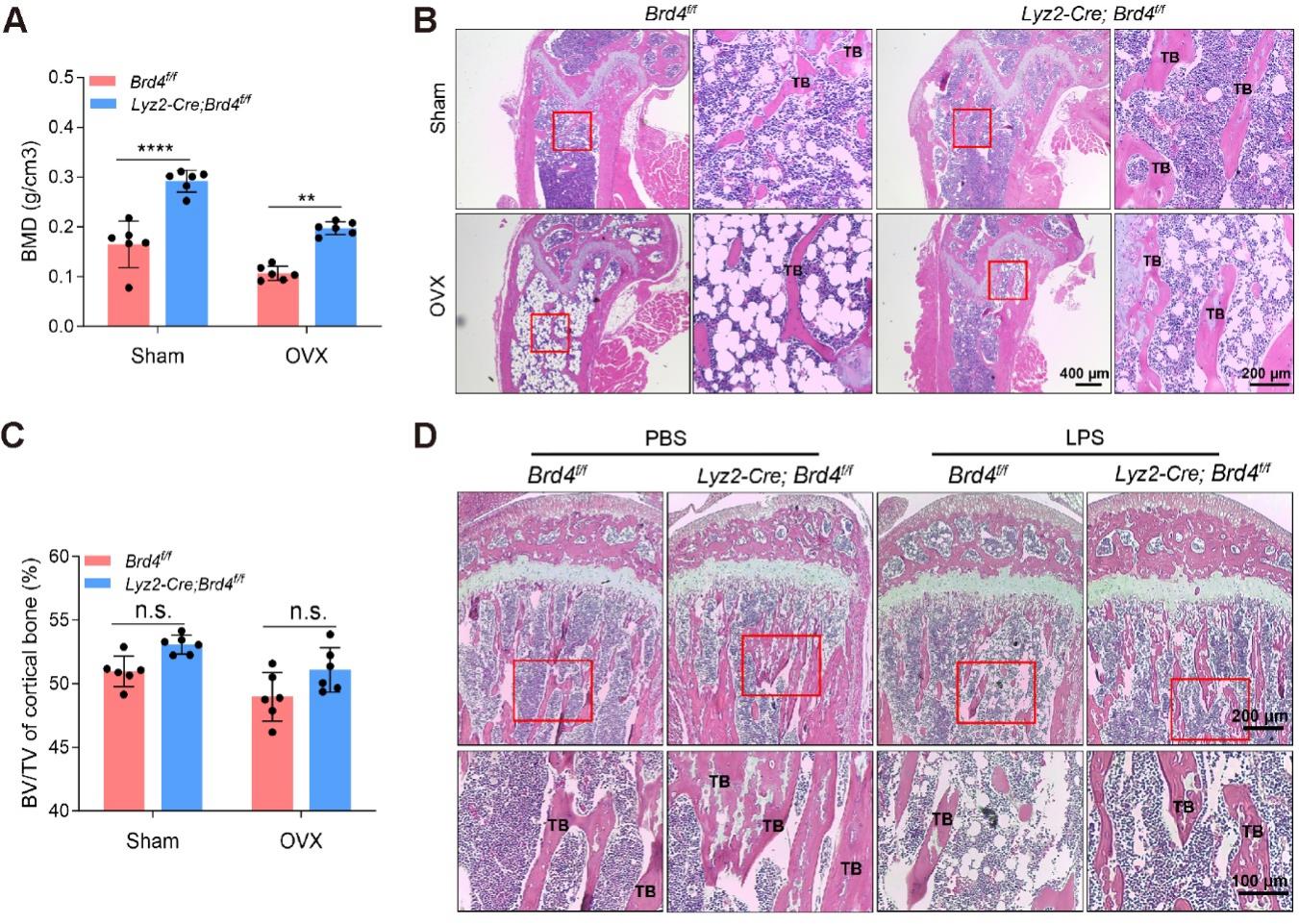


**Figure S6** **Depletion of *Brd4* in OC progenitors protects mice from pathological bone loss.** **A, C** Micro-CT analysis (BMD and BV/TV of cortical bone) of the distal femur of *Brd4^f/f^* and *Lyz2-Cre; Brd4^f/f^* mice induced by sham or OVX surgery (n = 6). **B** Representative H&E staining images of femur sections in various groups. Regions in the red square insets were enlarged in the corresponding right panel. **D** Representative H&E staining images of distal femur sections in various groups. The regions in the red square inset were enlarged in the corresponding below panel. Comparisons were conducted by one-way ANOVA analyses. ***p* < 0.01, *****p* < 0.0001, n.s., not significant.


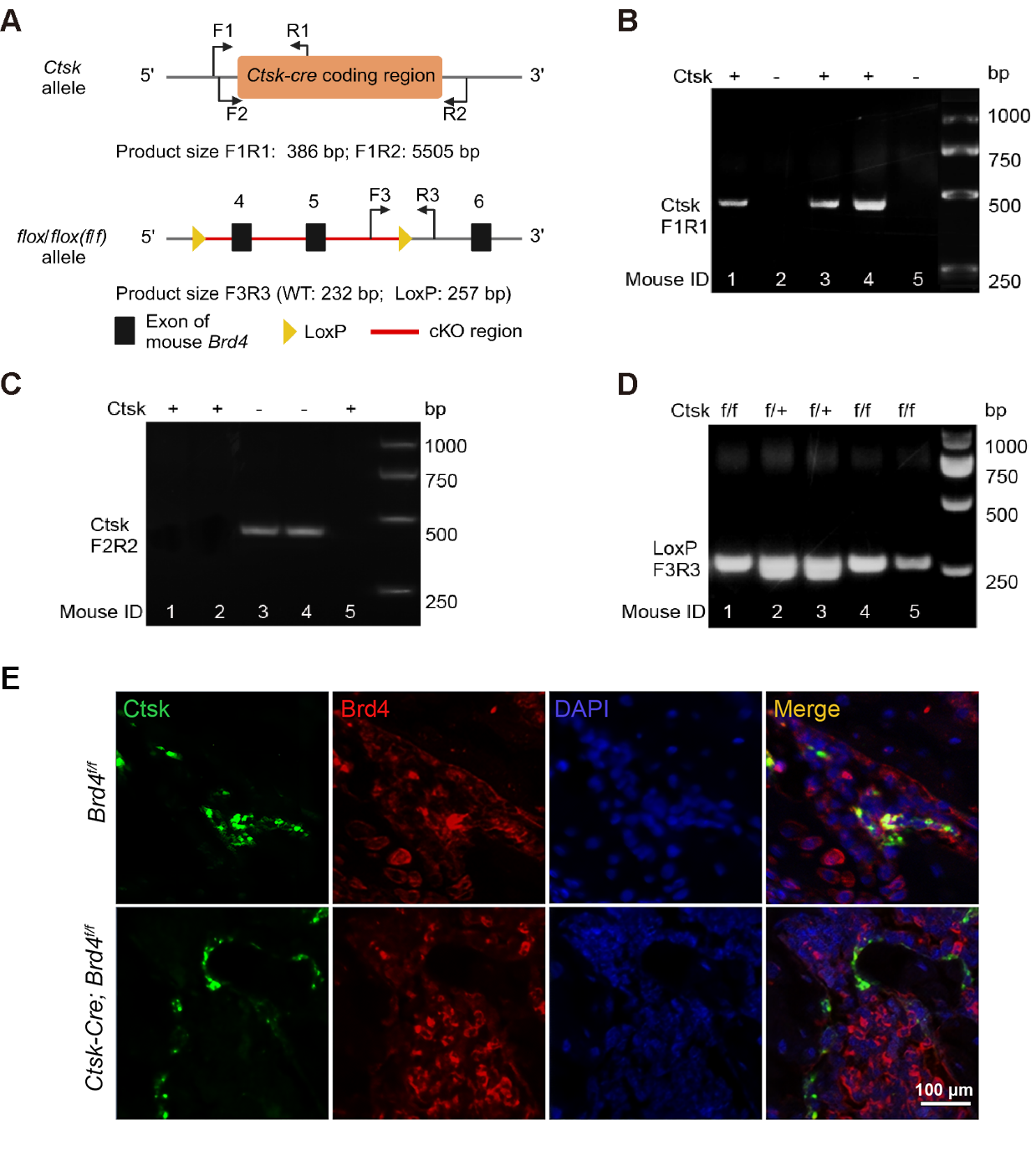


**Figure S7** **Establishment and validation of OC *Brd4* conditional knockout mice.** **A** Genotyping of *Ctsk-Cre; Brd4^f/f^* and *Brd4^f/f^* mice by RT-PCR. **B to D** Genotypic validation of *Brd4^f/f^* and *Ctsk-Cre; Brd4^f/f^* mice by RT-PCR. The genotypes of the five mice are *Ctsk^+/+^; Brd4^f/f^, Ctsk^-/+^; Brd4^f/+^, Ctsk^+/-^; Brd4^f/+^, Ctsk^+/-^; Brd4^f/f^, and Ctsk^-/+^; Brd4^f/f^*, respectively. **E** Knockout efficiency of Brd4 in BMMs was evaluated via immunofluorescence.


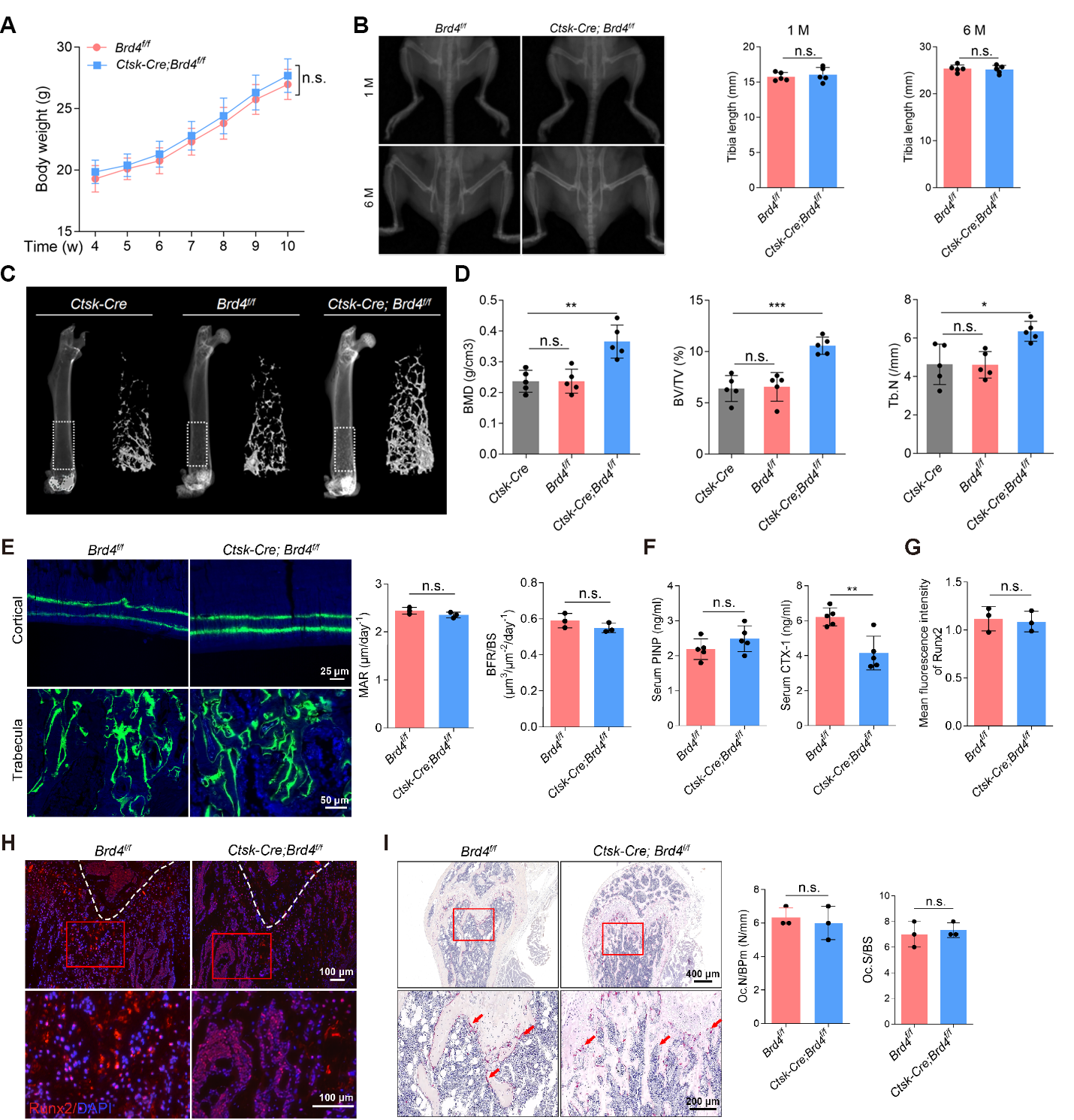


**Figure S8** **Deletion of *Brd4* in OC leads to high bone mass. A** Growth curve showing the body weight gained between *Brd4^f/f^* and *Ctsk-Cre; Brd4^f/f^* mice from 4 to 10 weeks of age. **B** X-ray images of *Brd4^f/f^* and *Ctsk-Cre; Brd4^f/f^* mice at 1 and 6 months old (left). Quantitative analysis of the tibia length of mice at different ages (right) (n = 5). **C** Representative micro-CT images of the femur of 12-week-old mice with different genotypes. **D** Quantitative measurements of BMD, BV/TV, and Tb.N (n = 5). **E** Visualization of double calcein labeling in *Brd4^f/f^* and *Ctsk-Cre; Brd4^f/f^* mice at 4 weeks of age (left), with quantification of MAR and BFR/BS (right) (n = 3). **F** ELISA detection of PINP and CTX-1 in serum of 8-week-old mice (n = 5). **G, H** Representative immunofluorescence images (H) and quantitative analysis of Runx2 (G) (red) in the distal femur sections of *Brd4^f/f^* and *Ctsk-Cre; Brd4^f/f^* mice at 8 weeks of age. White dashed lines outline the growth plate of the distal femur (n = 3). **I** Representative images of TRAP staining in the distal femur of 8-week-old *Ctsk-Cre; Brd4^f/f^* mice and control littermates (left), with the quantification of OC.N/BPm and OC.S/BS (right) (n = 3). Comparisons were conducted by Student’s t test, two-tailed. **p* < 0.05, ***p* < 0.01, ****p* < 0.001, n.s., not significant.


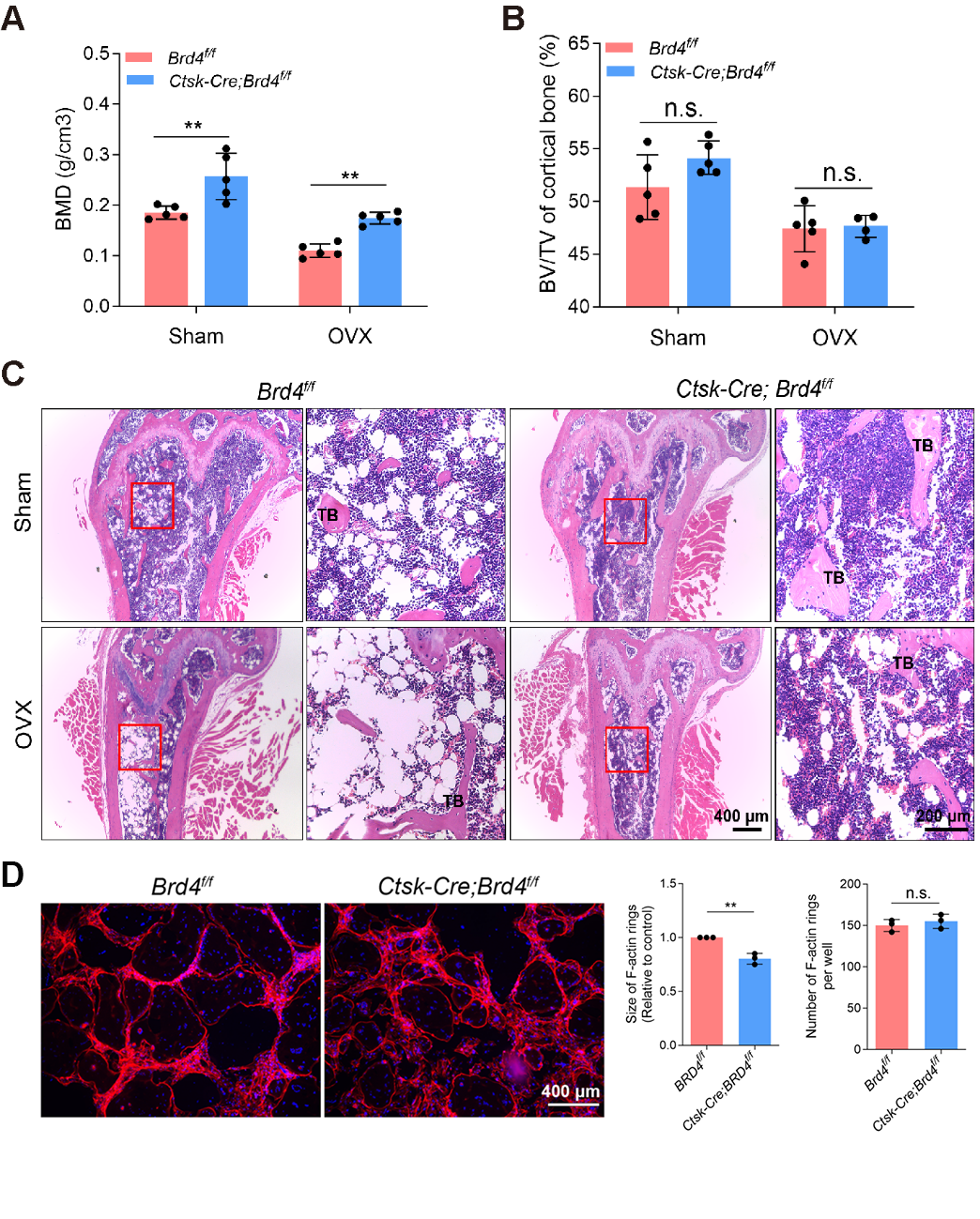


**Figure S9** **Loss of *Brd4* in OC protects mice from pathological bone loss. A, B** Micro-CT analysis of BMD and BV/TV in the cortical bone of the distal femur of *Brd4^f/f^* and *Lyz2-Cre; Brd4^f/f^* mice underwent sham or OVX surgery (n = 5). **C** Representative H&E staining images of the distal femur sections in various groups. The regions in the red square inset were enlarged in the corresponding right panel. Comparisons were conducted by one-way ANOVA analyses. ***p* < 0.01, n.s., not significant. **D** Representative images of F-actin staining (left) and quantification analysis (right) of BMMs from 8-week-old Ctsk-Cre; Brd4f/f mice and their control littermate after 5 days of OC induction (n = 3).


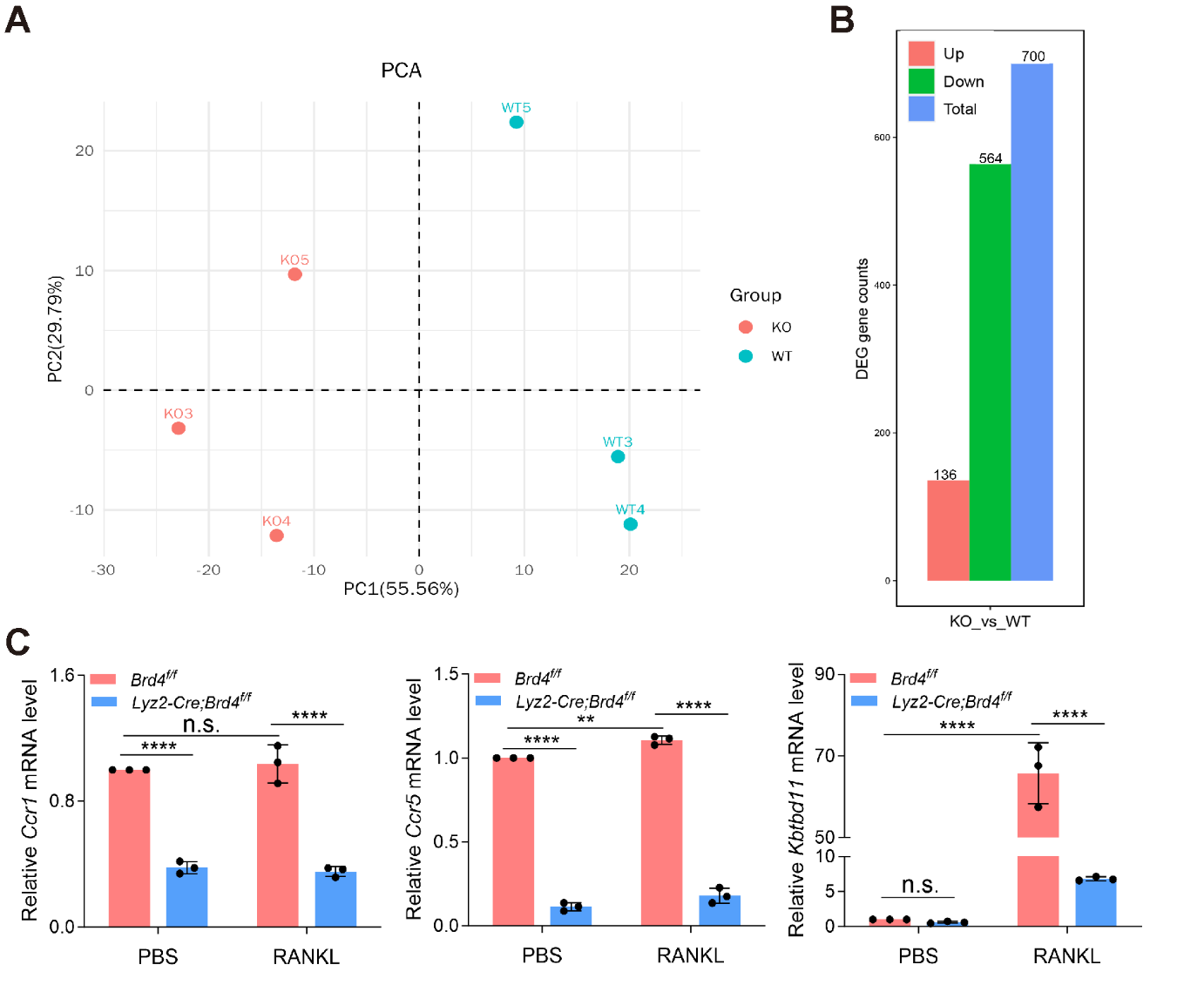


**Figure S10** **Identification of Brd4 target genes using RNA-seq. A** Principal component analysis (PCA) of the variance-stabilized estimated raw counts of DEGs. **B** Schematic diagram illustrating the downregulated and upregulated (|log_2_ (fold change)| > 1.0, p < 0.05) DEGs identified in the BMMs of *Lyz2-Cre;* *Brd4^f/f^* mice relative to their control littermates. **C** qRT-PCR analysis of the mRNA level of *Ccr1*, *Ccr5*, and *Kbtbd11* in BMMs treated with or without RANKL (n = 3). Comparisons were conducted by one-way ANOVA analyses. ***p* < 0.01, *****p* < 0.0001, n.s., not significant.


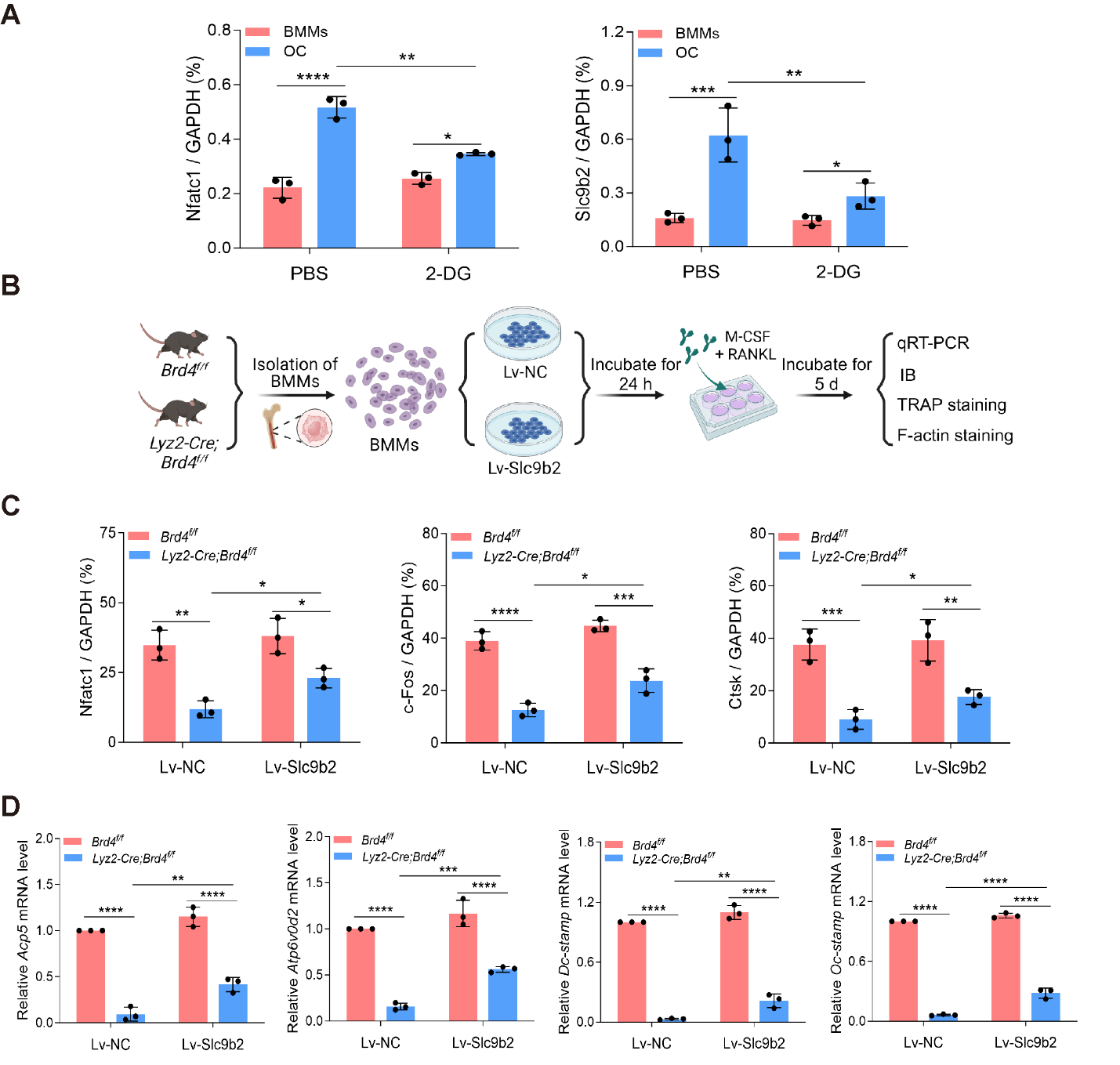


**Figure S11** **Overexpression of Slc9b2 partially rescues the Brd4-suppressed OC differentiation.** **A** Relative quantification of the protein expression levels of Nfatc1 and Slc9b2 in BMMs treated with OC induction and 2-DG or not (n = 3). **B** Schematic diagram illustrating the isolation of BMMs, infection with lentivirus, and OC analysis. **C** Relative quantification of the protein expression levels of Nfatc1, c-Fos, and Ctsk (n = 3). **D** qRT-PCR analysis of *Acp5*, *Atp6v0d2*, *Dc-stamp,* and *Oc-stamp* mRNA expression in BMMs with or without OC induction (n = 3). Comparisons were conducted by one-way ANOVA analyses. **p* < 0.05, ***p* < 0.01, ****p* < 0.001, *****p* < 0.0001.

**
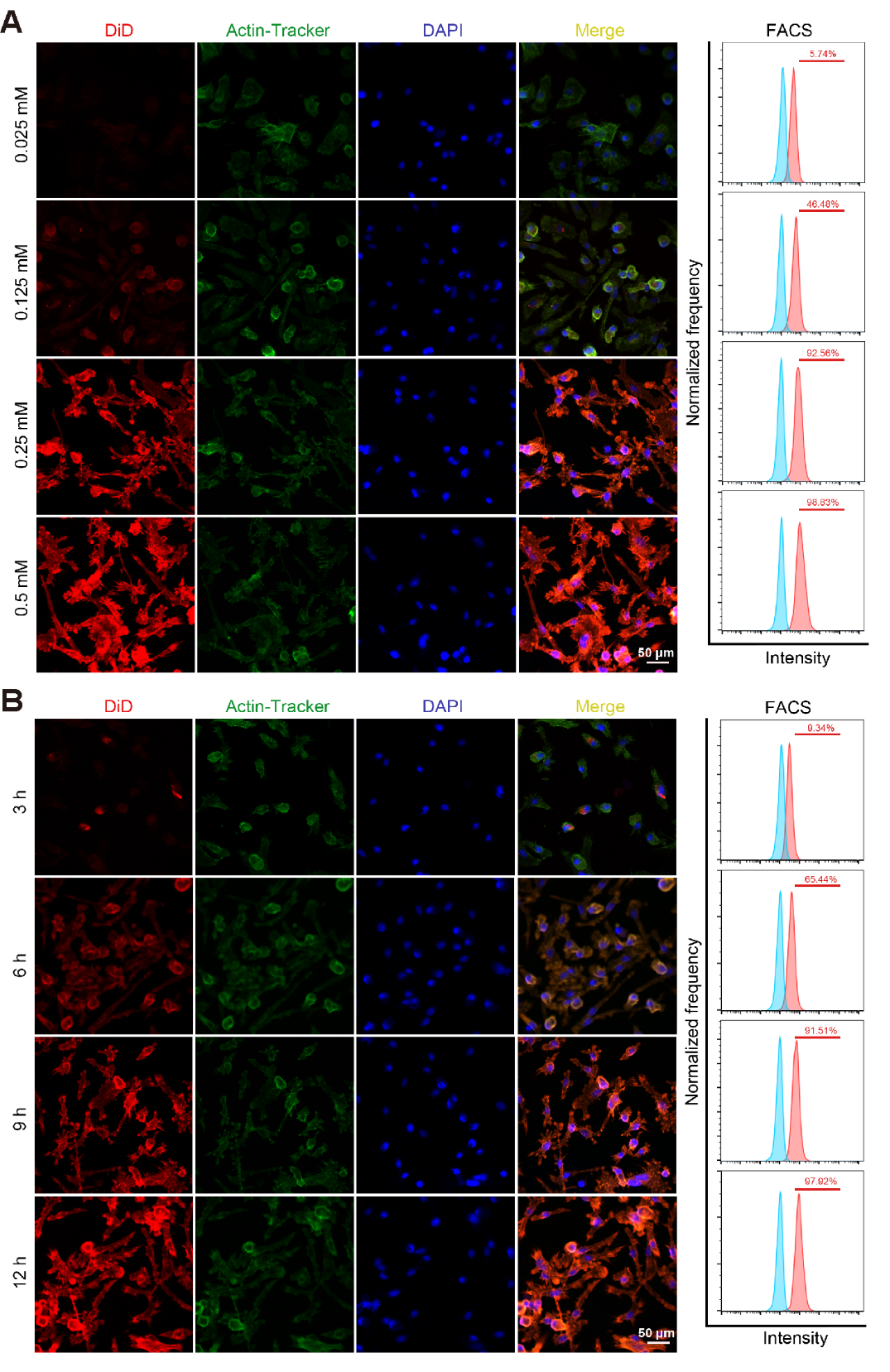
**

**Figure S12** **Cellular uptake of dBET6@PSLs/DiD by BMMs. A** Dose-dependent uptake of different formulations by BMMs, as determined by CLSM (left) and FACS (right). BMMs were treated with various concentrations of dBET6@PSLs/DiD (dBET6 equivalent to 10, 50, 100 and 200 nM) for 9 h. **B** Time-course analysis of uptake of different formulations by BMMs, as determined by CLSM (left) and FACS (right). BMMs were exposed to dBET6@PSLs/DiD (dBET6 equivalent to 100 nM) for 3, 6, 9 and 12 h.


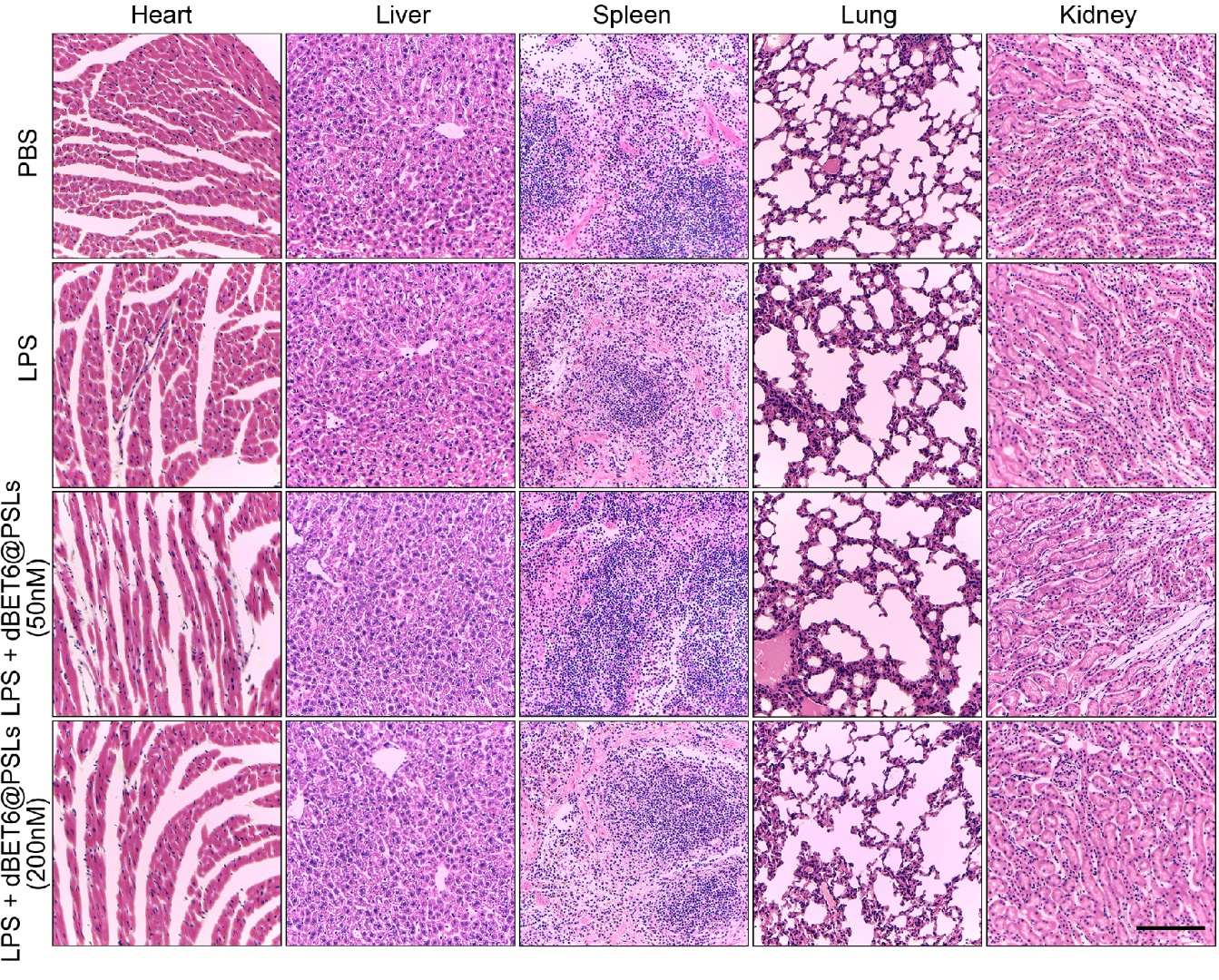


**Figure S13** **T****oxicity of dBET6@PSLs in mice. Representative** **H&E staining images of the major organs in various groups.**


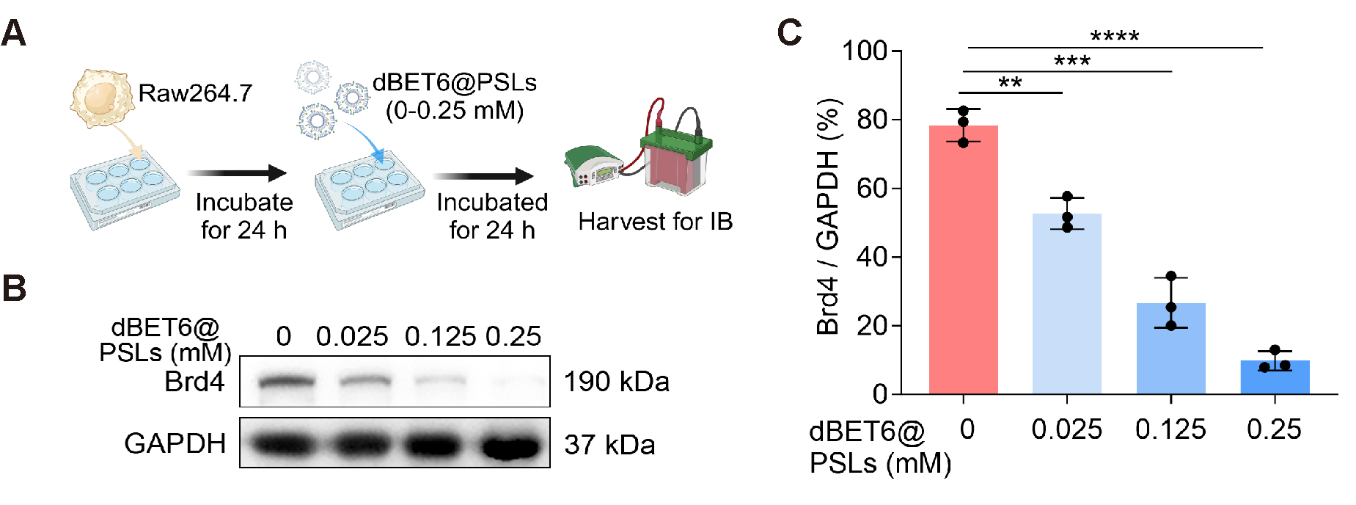


**Figure S14 The degradation efficiency of Brd4 by dBET6@PSLs *in vitro*. A** Schematic diagram illustrating the efficiency of Brd4 degradation in raw264.7 by dBET6@PSLs. **B,C** Validation of Brd4 degradation by dBET6@PSLs in raw264.7 cells with immunoblotting analysis (n = 3). Comparisons were conducted by Student’s t test, two-tailed. **p < 0.01, ***p < 0.001, ****p < 0.0001.


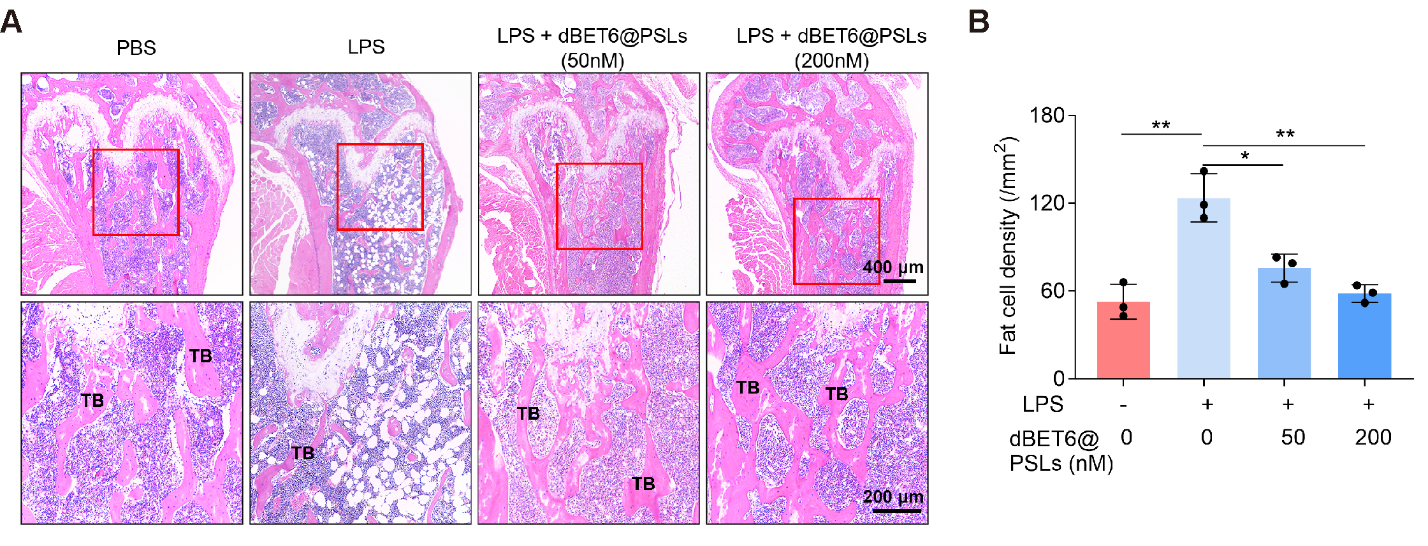


**Figure S15** **dBET6@PSLs reduces fat cell density.** **A, B** Representative H&E staining images and quantitative analysis of femur sections in the 12-week-old WT mice treated with 50 or 200 nM dBET6@PSLs in the presence or absence of LPS induction (n = 3). Comparisons were conducted by Student’s t test, two-tailed. **p* < 0.05; ***p* < 0.01.

Table S1. A detailed information of each patient enrolled for analysis


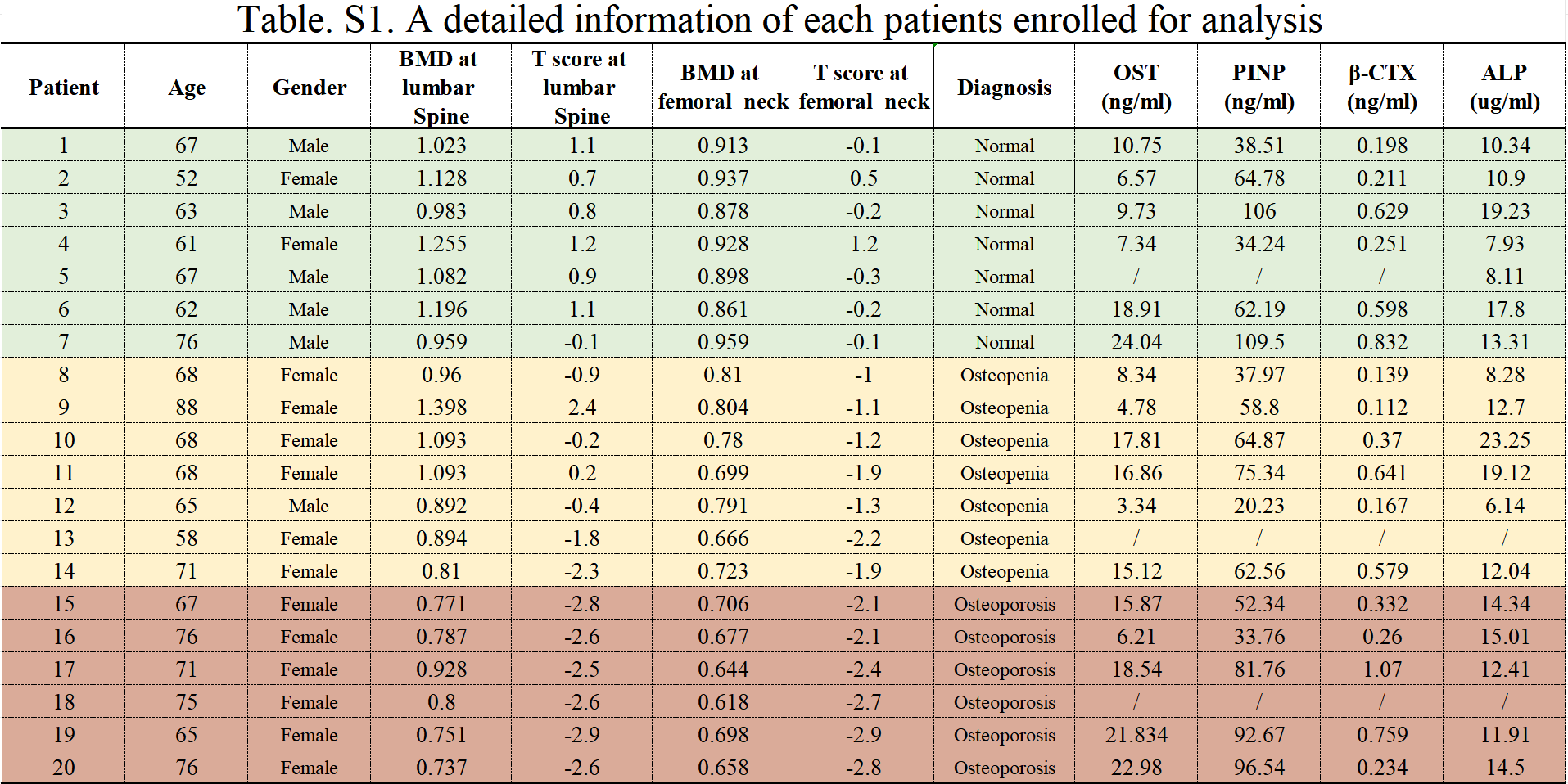


**Table S2.** Change of size, zeta potential, and PDI of dBET6@PSLs incubated with 10% FBS at various times

| **Time (h)** | **Size (nm)** | **PDI** | **Zeta potential (mV)** |
| --- | --- | --- | --- |
| 0 | 129.1 ± 1.6 | 0.261 ± 0.015 | -30.3 ± 0.9 |
| 0.5 | 136.0 ± 1.3 | 0.276 ± 0.005 | -27.5 ± 1.2 |
| 1 | 136.3 ± 0.9 | 0.300 ± 0.025 | -27.4 ± 1.8 |
| 6 | 140.6 ± 3.7 | 0.277 ± 0.015 | -26.4 ± 1.7 |
| 12 | 148.3 ± 0.8 | 0.257 ± 0.035 | -25.8 ± 2.1 |
| 24 | 149.7 ± 0.1 | 0.269 ± 0.011 | -26.7 ± 1.4 |
| 36 | 149.5 ± 3.5 | 0.268 ± 0.009 | -27.7 ± 1.5 |
| 48 | 155.6 ± 1.1 | 0.269 ± 0.005 | -26.6 ± 1.6 |

Table S3. Primers used for Brd4 conditional knock-out mice genotyping

| **siRNA** | **Forward** | **Reverse** |
| --- | --- | --- |
| *Lyz2-Cre*  *F1, R2* | AGTGCTGAAGTCCATAGATCGG | CTGATTCTCCTCATCACCAGG |
| *Lyz2-Cre*  *F1, R2* | AGTGCTGAAGTCCATAGATCGG | GTCACTCACTGCTCCCCTGT |
| *Ctsk-Cre*  *F1, R1* | GGGCAGTCTGGTACTTCCAAGCT | GAGCCCAGATCCACATCTGAACTG |
| *Ctsk-Cre*  *F1, R2* | CAGCAAAACCTGGCTGTGGATC | ATGAGCCACCATGTGGGTGTC |
| *loxP-F3, R3* | GTATTTCACCATTGAGCTTCATGCC | TGGCTGCTTGAGTTTTGTTAGTAGT |

**Table S4.** The primers used for quantitative RT-PCR

| **Genes** | **Forward** | **Reverse** |
| --- | --- | --- |
| *GAPDH* | TGTGTCCGTCGTGGATCTGA | TTGCTGTTGAAGTCGCAGGAG |
| *Brd4* | TCGTCTTAATGGCAGAAGCT | GTTTCTTTCCTCCCTCGTCC |
| *h-BRD4* | GCTTCAGGGTCTCAAAGTCG | GCTCAGCTTGGACATCAACA |
| *Nfatc1* | AGCTGCGCAGAATGAGATG | CAGTTTTGCCAAGGAGTGCT |
| *Acp5* | GTTTTCCTTGGGGTCCAGAC | CCAGACCAAGGTCAACCTCC |
| *Atp6vod2* | CAGACTCGGCAAAGTCGAG | CGACTGGACGAGAGGGATTG |
| *Mmp9* | GGAGAGCACATTCACGGTC | GATCTCAGTGCAGAGGCTCG |
| *Ctsk* | TTTGCTTGTCCAGGTGGTCC | GGAGCACTCTGTGTGTGCAA |
| *Slc9b2* | ATCCAACACAAGTGGTCGTC | GCCAGCACGAACCAGAATGA |
| *Kbtbd11* | CCAGCCCTTTCGTTGTGTTG | CTGCCACCAGGTCCCATATC |
| *Ccr1* | GCCGTGCGTCTGATATTTGC | CACAACAGTGGGTGTAGGCA |
| *Ccr5* | TTCTGGGCTCACTATGCTGC | TCACCCCAAAGTTGACCGTT |
| *H1f2* | CGTCTAAAGCCGTAAAGCCA | GAGCCTTTTGGATTGTTAGGG |
| *Oc-stamp* | ACAGCCCAGCCTAAGTTGTC | AAGAGCAATGCCAGTAGCCC |
| *Dc-stamp* | CCTGGTTCGAGGATGCAAAG | GGTCTCACACTGCACTAGGT |
| *Runx2* | CACTACCCAGCCACCTTTAC | AGGATGCTGACGAAGTACCA |
| *Osterix* | CCAGGCAACACACCTACTCC | GGGAGCAAAGTCAGATGGGT |
| *Alp* | TGGACGGTGAACGGGAAAAT | CATACGCCATCACATGGGGA |

**Reference**

1. Liu RX, Gu RH, Li ZP, Hao ZQ, Hu QX, Li ZY, et al. Trim21 depletion alleviates bone loss in osteoporosis via activation of YAP1/beta-catenin signaling. Bone Res 2023;11(1):56.

2. Yang J, Li S, Li Z, Yao L, Liu M, Tong KL, et al. Targeting YAP1-regulated Glycolysis in Fibroblast-Like Synoviocytes Impairs Macrophage Infiltration to Ameliorate Diabetic Osteoarthritis Progression. Adv Sci (Weinh) 2024;11(5):e2304617. [eng].
